# Neurohumoral Cardiac Regulation: Optogenetics Gets Into the Groove

**DOI:** 10.3389/fphys.2021.726895

**Published:** 2021-08-31

**Authors:** Arianna Scalco, Nicola Moro, Marco Mongillo, Tania Zaglia

**Affiliations:** ^1^Department of Cardiac, Thoracic, Vascular Sciences and Public Health, University of Padova, Padova, Italy; ^2^Veneto Institute of Molecular Medicine, Padova, Italy; ^3^Department of Biomedical Sciences, University of Padova, Padova, Italy

**Keywords:** optogenetics, autonomic neurons, heart innervation, brain–heart axis, neurogenic heart control

## Abstract

The cardiac autonomic nervous system (ANS) is the main modulator of heart function, adapting contraction force, and rate to the continuous variations of intrinsic and extrinsic environmental conditions. While the parasympathetic branch dominates during rest-and-digest sympathetic neuron (SN) activation ensures the rapid, efficient, and repeatable increase of heart performance, e.g., during the “fight-or-flight response.” Although the key role of the nervous system in cardiac homeostasis was evident to the eyes of physiologists and cardiologists, the degree of cardiac innervation, and the complexity of its circuits has remained underestimated for too long. In addition, the mechanisms allowing elevated efficiency and precision of neurogenic control of heart function have somehow lingered in the dark. This can be ascribed to the absence of methods adequate to study complex cardiac electric circuits in the unceasingly moving heart. An increasing number of studies adds to the scenario the evidence of an intracardiac neuron system, which, together with the autonomic components, define a little brain inside the heart, in fervent dialogue with the central nervous system (CNS). The advent of optogenetics, allowing control the activity of excitable cells with cell specificity, spatial selectivity, and temporal resolution, has allowed to shed light on basic neuro-cardiology. This review describes how optogenetics, which has extensively been used to interrogate the circuits of the CNS, has been applied to untangle the knots of heart innervation, unveiling the cellular mechanisms of neurogenic control of heart function, in physiology and pathology, as well as those participating to brain–heart communication, back and forth. We discuss existing literature, providing a comprehensive view of the advancement in the understanding of the mechanisms of neurogenic heart control. In addition, we weigh the limits and potential of optogenetics in basic and applied research in neuro-cardiology.

## Preface

From a simplistic point of view, the heart is a muscular pump which powers blood in the circulation, supplying oxygen, nutrients, hormones, and other tissue-derived mediators to all cells in the body (Braunwald et al., 1967). The initial observation that automaticity of heart contractions was independent from neuronal inputs, dates back to the second century, when Galen noted that “the fact that the heart, removed from the thorax, can be seen to move for a considerable time, is a definite indication that it does not need the nerves to perform its own function” (Charles, 1956). Throughout the more recent history of physiology, the concept of the heart as a strictly “muscular self-operating organ” was sometimes disputed, but did finally consolidate into a rock-solid notion with the cellular and molecular characterization of cardiac pacemaker and conduction system cells, still unanimously agreed (Keith and Flack, 1906; Tawara, 1906; Wybauw, 1910; Lewis, 1911; James and Sherf, 1971; Monfredi et al., 2010; Padala et al., 2021). It is undoubted that the explanted heart, if perfused with an appropriate solution, continues pumping while harvested from the organism (Ringer, 1883), and transplant of a heart disconnected from higher nervous components is a common life-saving procedure for patients with e.g., cardiac failure (McCartney et al., 2017).

It is, however, well-understood that the activity of the cardiac pump is finely tuned to match blood supply with the varied perfusional demand of the organism, e.g., increase of cardiac output during exercise to fuel skeletal muscle requirements (Higginbotham et al., 1986). Such extrinsic modulation of heart contraction is primarily operated by the sympathetic and parasympathetic branches of the autonomic nervous system (ANS), which innervate the atrial (both branches) and ventricular [mainly sympathetic neurons (SNs)] myocardium, exerting grossly opposite effects on the frequency and force of contraction (Zaglia and Mongillo, 2017). While the full-blown activation of SNs, easily detectable, suggests the dominant effect of the sympathetic branch of the ANS on heart control, preclinical and clinical evidence underscores the regulatory power of the parasympathetic nervous system (PNS) on heart function. While the remainder parts of this review will mainly focus on SNs, the reader is referred to Li et al. (2004) Lee et al. (2016), and Zasadny et al. (2020) for reference.

**Figure 1:**
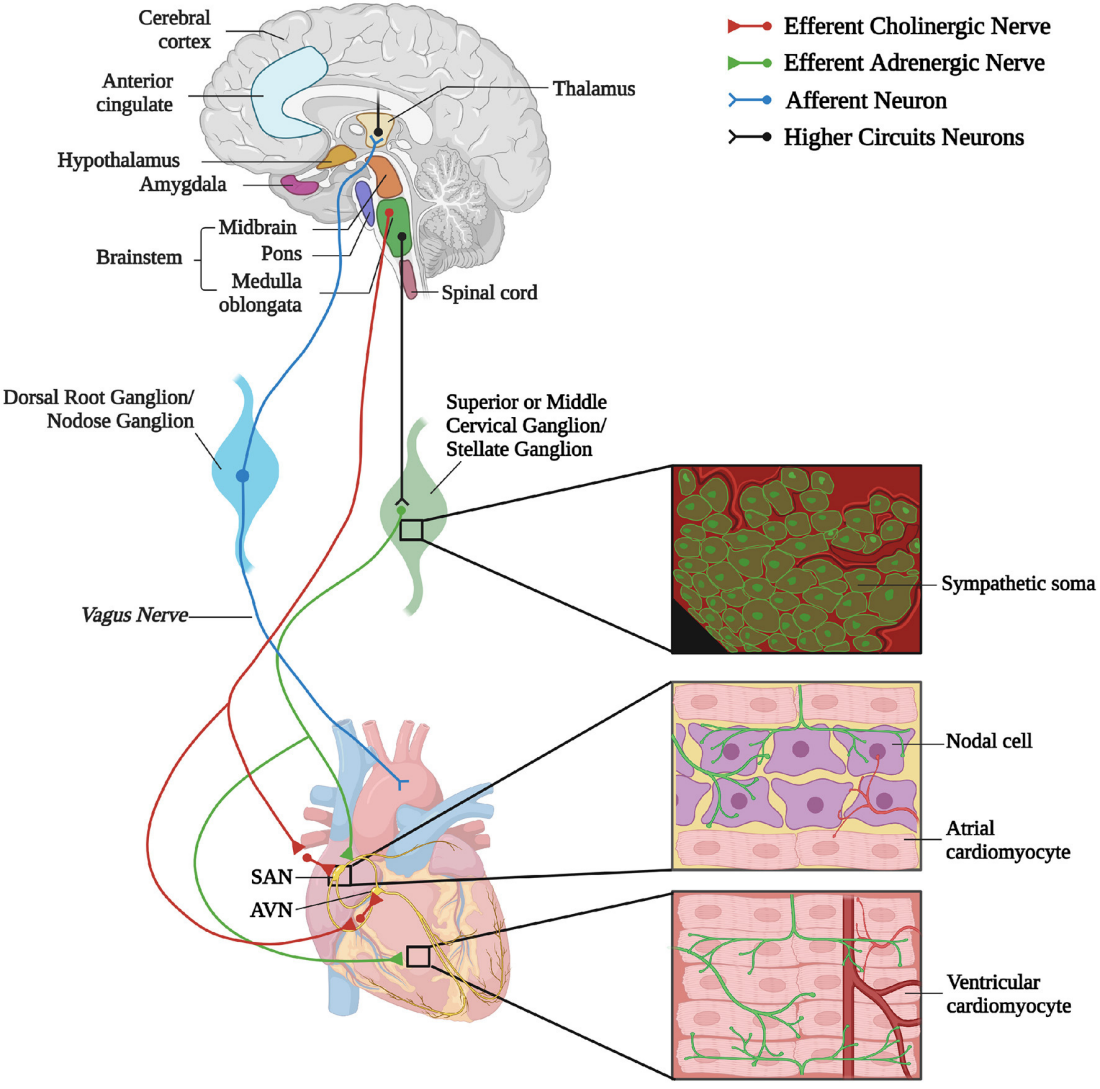
The complex neuronal circuitries underlying bidirectional “brain–heart” connection. Schematic representation of the “brain–heart axis.” Different regions of the brain, belonging to the CAN process precise orders which are transmitted, through efferent preganglionic fibers, to both sympathetic (adrenergic) and parasympathetic (cholinergic) cardiac ganglia. While PSN processes mainly innervate the SAN and the AVN, SNs invade the conduction system and the working myocardium. The cardiac muscle is also innervated by intrinsic neurons (INS) and cardiac sensory neurons, whose cell bodies organize into the dorsal root ganglion and nodose ganglion, and their afferent fibers project to different areas of the brain (created with BioRender.com).

A reductionist example of neurogenic heart regulation by SNs is shown by the effect of a decrease in blood pressure, which is immediately perceived by peripheral baroceptors, and reflects centrally on the activation of sympathetic nervous system (SNS), increasing heart rate (HR), contraction force, and peripheral vasoconstriction, to restore the physiologic pressure values (Heesch, 1999; Fadel, 2008). Beyond such rather unsophisticated, single lane neuro-cardiac connection, however, the activity of cardiac autonomic neurons is orchestrated by a complex network of neural circuits established in the central nervous system (CNS) [i.e., the central autonomic network (CAN)], which includes various regions in the cortex, amygdala, hypothalamus, in the midbrain and pons, and in several nuclei in the medulla (Silvani et al., 2016). These stations receive information from other parts of the brain (e.g., emotional inputs), peripheral body sensors (i.e., homeostatic inputs), as well as from the external environment (i.e., sensory inputs), all of which are integrated to come up with an appropriate response, acutely dictated to the heart through preganglionic efferents converging on the sympathetic and parasympathetic ganglia (Ardell and Armour, 2016; Silvani et al., 2016). Post-ganglionic “motor” neurons, in turn, divaricate from the ganglia toward appropriate sections of the heart and blood vessels, delivering the operative information to the cardiovascular system (Franzoso et al., 2016; Di Bona et al., 2020) (Figure 1).

In addition to a “loud” brain–heart communication in the “fight-or-flight” reaction, however, a whispered and perpetual neurocardiac dialogue, finalized to preserve the heart, and organism's homeostasis, has been demonstrated by a large number of studies, including ours (Ogawa et al., 1992; Kanevskij et al., 2002; O'Connell et al., 2003; Zaglia et al., 2012; Kreipke and Birren, 2015; Pianca et al., 2019). Constitutive activity of the ANS (both from the sympathetic and parasympathetic branches) regulates HR on a beat-to-beat basis (Ahmed et al., 1994; Lombardi et al., 1996; Poletto et al., 2011; Vanderlaan et al., 2012; Moreno et al., 2019) and controls, in parallel, signaling pathways impinging on cardiomyocyte (CM) proteostasis, size, electrophysiology, and division (Ogawa et al., 1992; Kanevskij et al., 2002; O'Connell et al., 2003; Zaglia et al., 2012; Kreipke and Birren, 2015; Pianca et al., 2019). Beyond cardiac physiology, the role of the “brain–heart axis” is increasingly being recognized in cardiac pathology, as highlighted by the widespread use of sympatholytic therapies (e.g., β-blockers) (Ponikowski et al., 2016; Ibanez et al., 2018; Neumann et al., 2020). Chronic sympathetic disturbances have a role in myocardial hypertrophy (Zimmer et al., 1995; Kimura et al., 2007; Fukuda et al., 2015) and heart failure (HF) (Kishi, 2012; Fukuda et al., 2015), and acute sympathetic hyperactivity, during acute physical exercise or following intense emotional stresses, is responsible for arrhythmias and sudden cardiac death (SCD) in several genetic cardiac disorders (e.g., Arrhythmogenic Cardiomyopathy, ACM; Catecholaminergic Polymorphic Ventricular Tachycardia, CPVT; Long QT Syndrome) (Leenhardt et al., 1995; Schwartz et al., 2001; Wehrmacher et al., 2005; Lehnart et al., 2008; Basso et al., 2009; Fukuda et al., 2015; Taggart et al., 2016; Corrado et al., 2017; Agrimi et al., 2020; Winbo and Paterson, 2020; Wleklinski et al., 2020), as well as in Takotsubo syndrome (Sharkey et al., 2011). In addition, it is now well-accepted that brain damage, by modifying autonomic control of heart function, reflects negatively on cardiac health [e.g., patients suffering a stroke have higher probability to develop secondary adverse cardiac events (Touzé et al., 2005; Kim et al., 2017)]. Along the same lines, cerebrovascular diseases may cause cardiac arrhythmias, potentially evolving in SCD (Mikolich and Jacobs, 1981; Kallmünzer et al., 2012; Ruthirago et al., 2016), and depression has been shown to increase the incidence of coronary artery disease (Bunker et al., 2003; Van der Kooy et al., 2007; Carney and Freedland, 2017) and heart attacks (Van der Kooy et al., 2007).

In parallel with the increased interest in the “brain-to-heart” communication, a number of studies unveiled that, besides a highly intricated network of post-ganglionic autonomic neurons (Janes et al., 1986; Kawashima, 2005; Zaglia and Mongillo, 2017; Wink et al., 2020), the myocardium homes an intrinsic nervous system (INS) (Armour et al., 1997; Fedele and Brand, 2020). In addition, afferent neurons may mediate the reverse “heart-to-brain” communication, by continuously sending information to the CNS, which impacts on neuronal circuits involved in perception, cognition, and emotional processing (Schievink et al., 2017; Dal Lin et al., 2018) (Figure 1).

When considering such complexity of the brain–heart neural wiring, the awareness emerges that brain-to-heart communication should not be simplified to the interaction between a brainy regulator and a heartly executor. The “little brain of the heart,” promoting this self-excitable muscle from mere hydraulic pump to sophisticated “neuro-muscular” organ (Armour, 2007, 2008), calls for its bright spot in the theater of physiology.

Traditional methodologies of neuroanatomy, neurophysiology, and pharmacology, which have commonly been used in neuro-cardiology, are inadequate to untangle the complex neuronal circuits linking the brain and the heart in an intimate bidirectional interaction. On the contrary, optogenetics represents the novel biotechnological tool which, by allowing spatially- and cell type-selective neuromodulation, has the potential to functionally dissect the components of the entire “brain–heart” circuitry, and resolve open questions on physiologic mechanisms which have remained obscure for a long time. This review will provide a description of the brain–heart connection and discuss how optogenetics has started to contribute shedding light on the unexpectedly intricated “neuro-cardiac liaison.”

## The Light Touch of Optogenetics

Optogenetics is a recently developed technology, which combines physics, molecular biology, and electrophysiology (Boyden et al., 2005; Deng et al., 2014) to enable contactless control of ion flux across the plasma membrane of cells exogenously expressing photoactivatable channel proteins consistently named “opsins.” The method, singled out as Nature's “*Method of the Year 2010*,” relies on the discovery that a microbial-derived class of proteins, with structural features resembling the well-known rhodopsins, form ion channels with variable selectivity, while retaining light sensitivity, two properties that allow to either depolarize or hyperpolarize the membrane potential of excitable cells with light irradiation at appropriate (i.e., depending on the opsin type) wavelength. The algal light-sensitive cation-conducting channelrhodopsins (ChRs) were among the first opsins to be characterized and expressed in a specific cell population, within a multicellular tissue of the experimental animal (Nagel et al., 2002, 2003; Zhang et al., 2008), to interrogate the role of specific neuronal circuits of the CNS. Two ChR variants have initially been used in neuroscience: ChR2 from *Chlamydomonas reinhardtii* (Boyden et al., 2005; Li et al., 2005; Nagel et al., 2005; Bi et al., 2006; Ishizuka et al., 2006; Zhang et al., 2006) and VChR1 from *Volvox carteri* (Zhang et al., 2008), endowed with sufficiently fast kinetics to achieve action potential (AP) triggered with brief light pulses (1–5 ms), delivered at a frequency suited to neuronal activation. For more detailed description on the history and development of optogenetics, the readers are referred to several topical reviews, some included in this same Special Issue (Hegemann and Nagel, 2013; ref to be included by editors).

The unique aspect which signed success of the technique, compared with conventional electrophysiological methods, based on electrical perturbation, are its non-invasiveness, spatial-temporal accuracy, and cellular specificity (Pianca et al., 2017; Sasse, 2018). The latter allowed to “interrogate” the function of a selected cell type while enclosed in a complex tissue, and intermingled with numerous different cells, giving neuroscientists the tools to selectively study a given brain region or even a single neural circuit (Rajasethupathy et al., 2016; Pianca et al., 2017). Moreover, the non-invasiveness of the technique allows to stimulate cells multiple times or for long periods, with no damage, an incredible advancement in experimental neuroscience, where such technology was firstly tested, and rapidly became a reference method (Pianca et al., 2017; Deubner et al., 2019).

The rocketing potential of optogenetics drove molecular biologists to expand the opsin variants toolkit, including newly identified (i.e., from other microbial species) and molecularly engineered opsins (for reference see: www.optogenetics.org). This led to the generation of a large number of opsin variants, endowed with specific ionic selectivity (e.g., for cations or anions) enabling either cell hyperpolarization [i.e., PAK-K (Bernal Sierra et al., 2018), BLINK-1 (Cosentino et al., 2015), GtACR1 (Govorunova et al., 2016), ArchT (Han et al., 2011), Halorhodopsin (Gradinaru et al., 2008), Jaws (Chuong et al., 2014)] or depolarization [i.e., ChR2 (Nagel et al., 2003), CheRiff (Hochbaum et al., 2014), ReaChR (Lin et al., 2013), Crimson (Klapoetke et al., 2014)], different photocurrent kinetics and sensitivity to differently-colored activation light, covering almost the entire visible spectrum, from blue- to red-shifted variants.

Some years after its initial application to neurosciences, the pioneering studies of Arrenberg (Arrenberg et al., 2010), Bruegmann (Bruegmann et al., 2010), and Jia (Jia et al., 2011) demonstrated that optogenetics can be applied also for the study of heart electrophysiology. In particular, Bruegmann and colleagues developed transgenic mice expressing ChR2 in CMs and were able to pace heart contractions by shining light on the epicardium, thus inducing ectopic heart beats *in vivo* (Bruegmann et al., 2010). Simultaneously, Arrenberg and colleagues combined the expression of Halorhodopsin and ChR2 in zebrafish CMs, with the use of light sheet microscopy, and achieved bidirectional control of HR *in vivo*, remotely inducing tachycardia, bradycardia and cardiac arrest (Arrenberg et al., 2010). In parallel, Jia et al. demonstrated “the utility of optogenetics to cardiac muscle by a tandem cell unit (TCU),” which “can serve not only as an elegant tool in arrhythmia research but may form the basis for a new generation of light-driven cardiac pacemakers and muscle actuators” (Jia et al., 2011).

**Figure 2:**
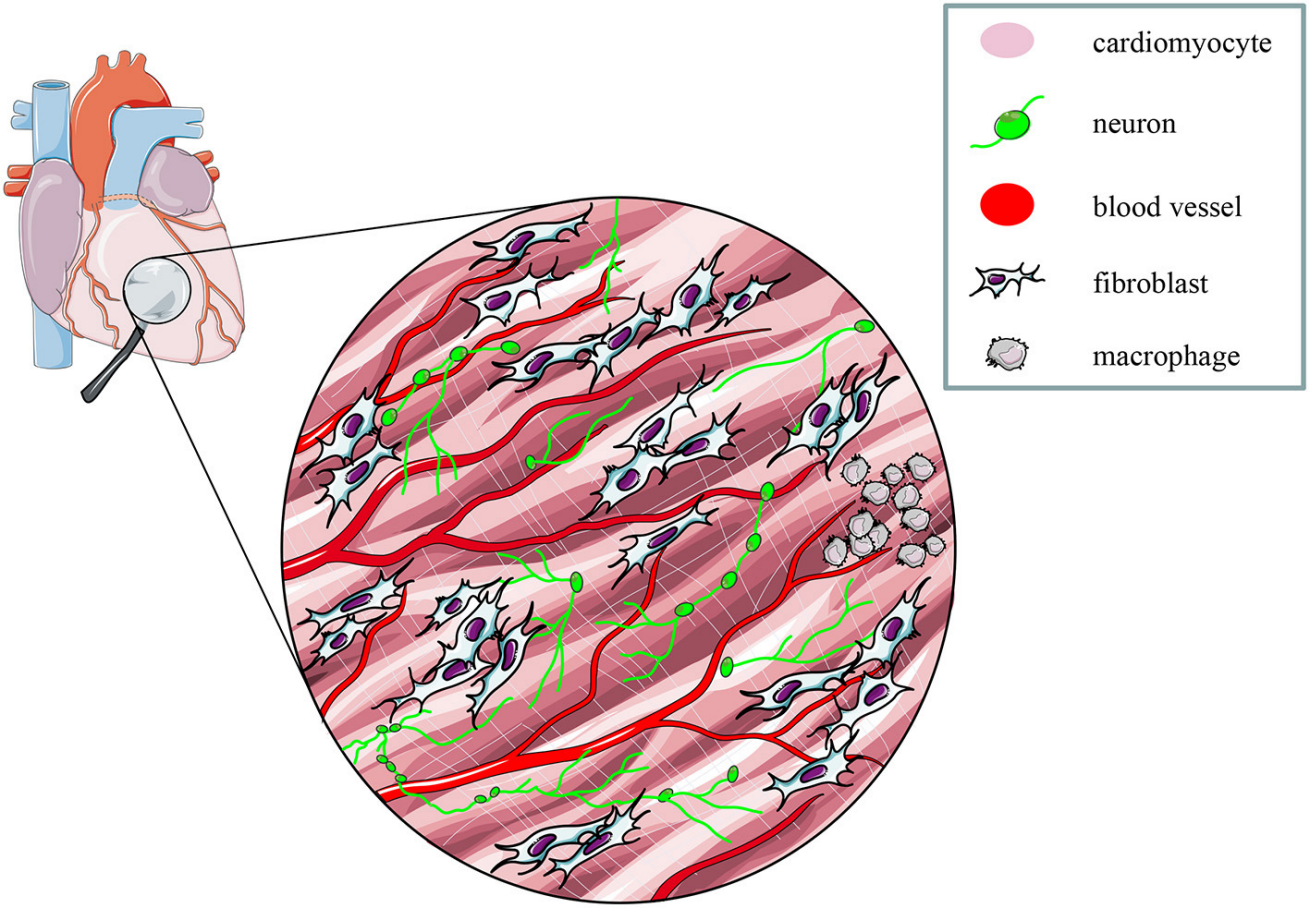
The multicellular nature of the myocardium. The myocardium is a complex network of different structural and functional interconnected cell types, including intrinsic and extrinsic excitable, as well as non-excitable cells. Modified with permission from Zaglia et al. (2019).

**Figure 3:**
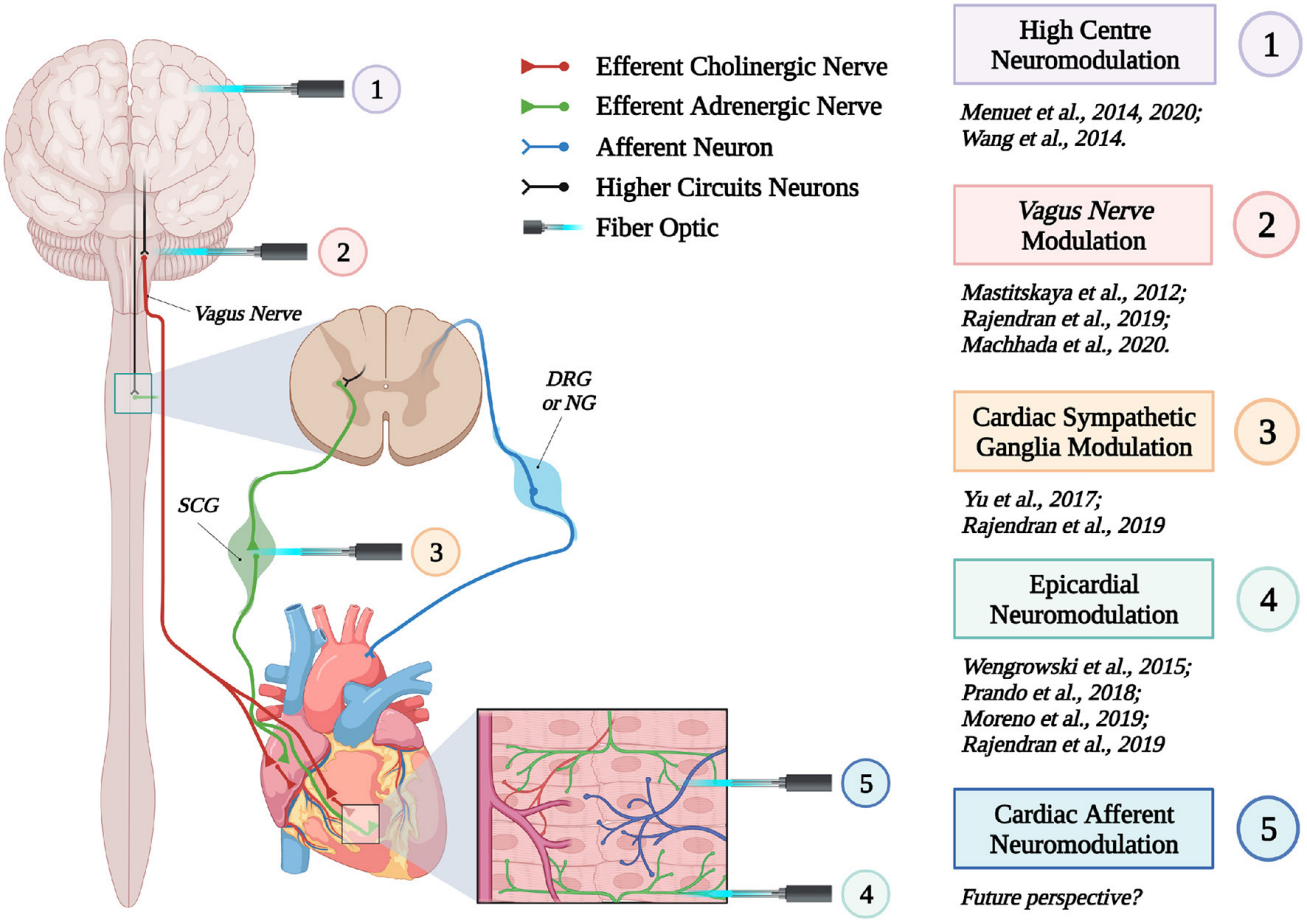
Optogenetic interrogation of the brain–heart axis at multiple sites. Schematic representation of the different brain–heart connecting circuitries which have been or could be interrogated by optogenetics (created with BioRender.com).

These proof-of concept experiments signed the beginning of “cardiac optogenetics,” which was subsequently applied to investigate biophysical aspects of cardiac physiology and pathology [i.e., minimal cell mass required to activate ectopic beats; role of Purkinje fibers (PFs) in arrhythmias (Zaglia et al., 2015; Pianca et al., 2017)]. Undoubtedly, these studies opened fascinating perspectives, but the inherent potential of cardiac optogenetics (i.e., cell type specificity and non-invasiveness) was uncovered by approaching the heart as a complex network of different structurally and functionally interconnected cell populations, including excitable (i.e., working CMs, conduction system cells, efferent, intrinsic, and afferent neurons), conducting (i.e., fibroblasts, resident, and recruited inflammatory cells), and non-excitable (i.e., vascular cells) cells (Pianca et al., 2017) (Figure 2). As heart homeostasis relies on the activity of many multicellular “circuitries,” fine dissection of the individual role of each cell type, with respect to localization and myocardial interactions, has benefitted, alike neurobiology, from optogenetic studies. Here, we will focus on heart-innervating autonomic neurons, and present examples of existing literature which describes how optogenetics allowed to: (i) improve the knowledge on the mechanisms underlying physiologic neurogenic control of heart function; (ii) investigate and lay new therapeutic concepts in arrhythmology, and (iii) add new tiles to the understanding of the bidirectional “brain–heart” interaction. In addition, we will comment on the opportunities that optogenetic-based neuromodulation of the heart could offer in the inspection of the multiplexed connections between brain and heart, from the central to the peripheral and heart intrinsic neuronal networks, impinging on heart function, in physiological and pathological contexts (Figure 3).

## Optogenetics Allows Untangle Cardiac Neuronal Circuitries

In June 2021, the simple query of “optogenetics” and “neuron” retrieves 5,700 hits in Pubmed, highlighting the aptness of the method in neuroscience research, and as such its use to interrogate the function of the cardiac neural network would thus appear a trivial task. Non-invasive optical actuation of heart neurons is a tremendously powerful technique, but it has to be considered that the spectrum of cardiac effects of acute neuro-cardiac modulation is rather limited. Granted, HR and contractile function can be monitored with standard methodologies (ECG, echocardiography), but study of e.g., heart electrophysiology at cellular level requires state-of-the-art methods to allow investigation *in vivo*. Thus, combination with equally advanced readouts of heart function is necessary, as discussed in more detail in **Chapter 6**.

### The Unexpected Extent of Cardiac Sympathetic Innervation

**Figure 4:**
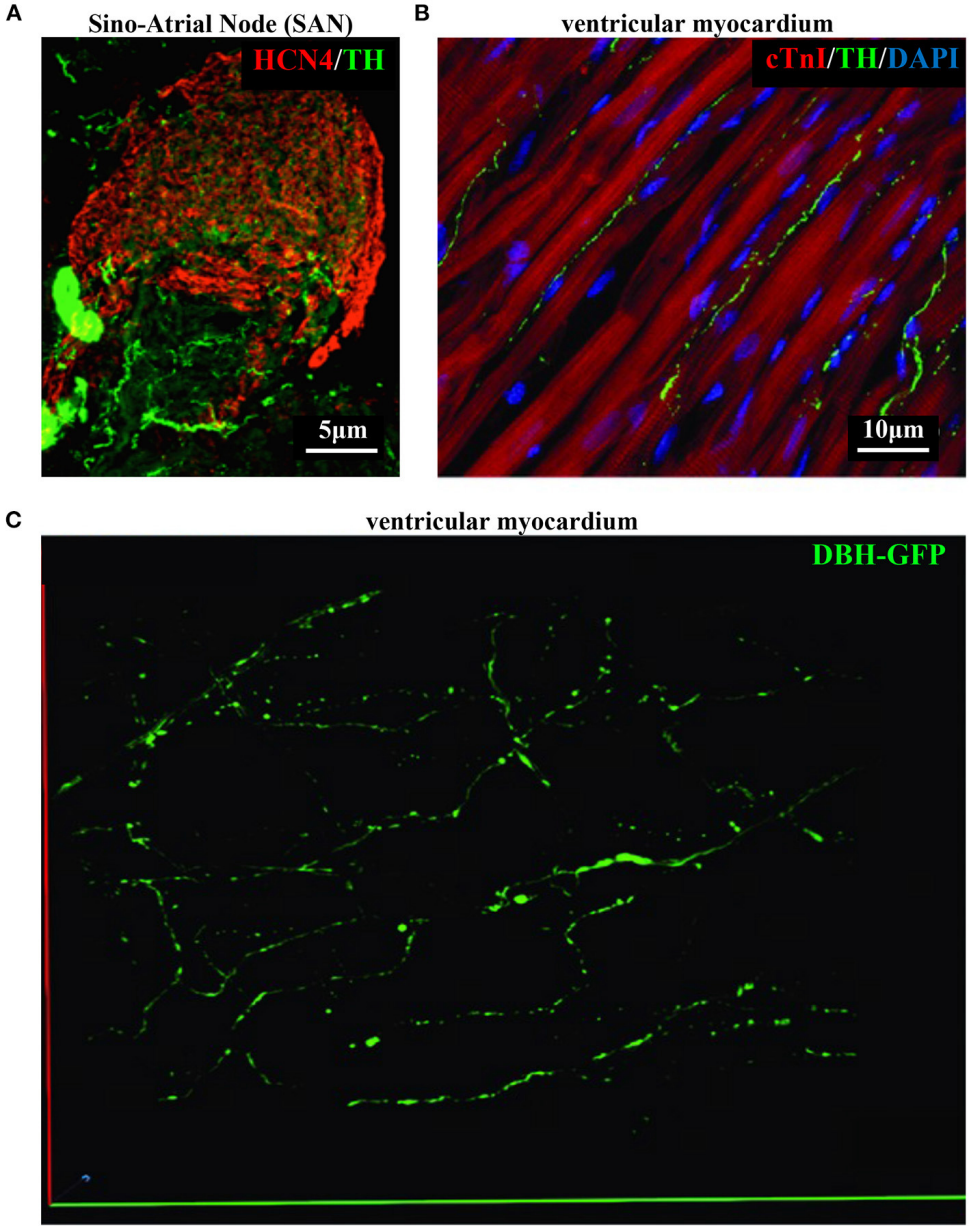
Bi- and three-dimensional topology of cardiac sympathetic innervation. **(A)** Confocal immunofluorescence of the SAN of a normal adult mouse, co-stained with antibodies to tyrosine hydroxylase (TH) and HCN4, to identify SN processes and pacemaker cells, respectively. **(B)** Confocal immunofluorescence imaging of ventricular myocardial section from normal adult mice, stained with antibodies to TH and cardiac troponin I (cTnI). Nuclei are counterstained with DAPI. The image is a detail from the LV subepicardial region. **(C)** 3-D reconstruction, at the multi-photon microscope of the sympathetic network within a portion of the LV subepicardium in an adult, Langendorff-perfused DBH-GFP heart. A segment of 230 μm by 28 μm by 50 μm was imaged. **(A–C)** Modified with permission from Prando et al. (2018) **(A)**, Zaglia and Mongillo (2017) **(B)**, and Freeman et al. (2014) **(C)**.

**Figure 5:**
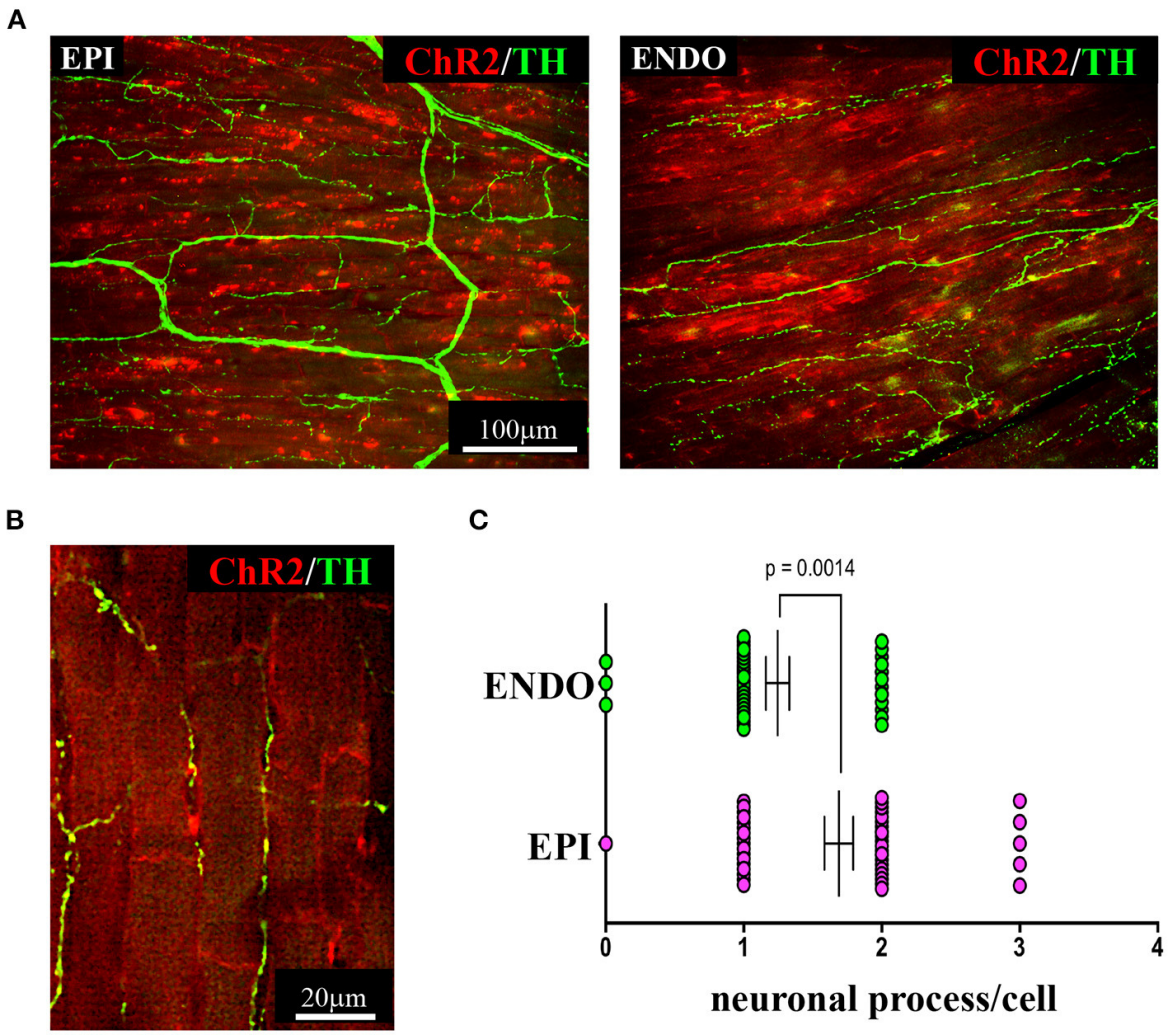
Three-dimensional imaging of the neuronal network in the murine myocardium. **(A)** Maximum intensity projection of multiphoton image stacks acquired along 400 μm in tissue clarified LV blocks from the EPI and ENDO regions of a α-MHC/ChR2-*td-Tomato* mouse heart, stained with an antibody to TH. Red emission of *td-Tomato* was used to identify CM membrane. **(B)** Representative single optical section of a sample processed as in **(A)**, resolving the neuro-cardiac interactions, and used for quantification of neuronal processes/cell in EPI and ENDO regions **(C)**. **(A–C)** Modified with permission from Pianca et al. (2019).

**Figure 6:**
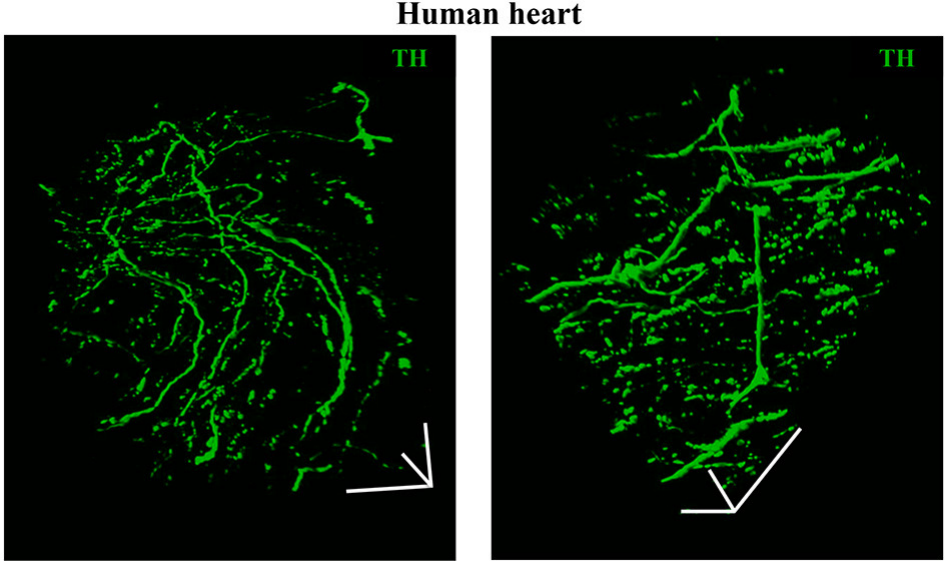
Three-dimensional imaging of the neuronal network in the human myocardium. Topology of the SN network, reconstructed with 3-D rendering of 600 images acquired with a multiphoton microscope along 200 μm, upon whole-mount immunofluorescence in 1 mm^3^ LV blocks from the EPI region, stained with anti-TH antibody. Images were acquired with an 18× objective, 1.1 NA, allowing a large field of view (850 × 850 μm) at high resolution. A segment of (850 × 850 × 200) μm was imaged. mage series were acquired along the Z-axis, with a step size of 1.5 μm and processed and analyzed with a software for 3-D rendering (Imaris). Images modified with permission from Pianca et al. (2019).

As discussed in **Chapter 1**, the parasympathetic and sympathetic branches of the ANS are both present in the myocardium, with parasympathetic neurons (PSNs) detectable in SAN, atrioventricular node (AVN), atria and cardiac blood vessels, and SNs innervating both the conduction system and the working myocardium of atria and ventricles (Franzoso et al., 2016) (Figures 4A,B). Heart innervation by the ANS has long been recognized in both physiology and diseases, as exemplified by the common use of β-adrenoceptor (β-AR) antagonists, inhibiting the main sympathetic neurotransmitter receptor, in several cardiovascular diseases (Zaglia and Mongillo, 2017). However, the precise anatomy, the extent and intercellular contacts of cardiac innervation have remained, quite surprisingly, understudied and elusive for a long time, partly due to technical limitations and methodologic difficulties. Here, we will mainly focus on SNs, whose processes display a “pearl necklace” morphology, characterized by regularly distributed varicosities, which are the neurotransmitter [i.e., mainly noradrenaline (NA) and neuropeptide Y (NPY)] releasing sites, contacting myocardial target cells (Zaglia and Mongillo, 2017) (Figures 4A,B). Characterization of SN topology was initially obtained by the analysis of thin rodent heart sections, stained with antibodies to enzymes involved in NA biosynthesis, which allowed to demonstrate that SNs heterogeneously distribute among the four heart chambers, with the highest density of nerve fibers in the atria, followed by the right (RV) and left (LV) ventricle (Di Bona et al., 2020). Moreover, across the ventricular walls a transmural gradient of SN density, from the epicardium to the endocardium can be detected in a specie-specific distribution in different mammalian hearts (Randall et al., 1968; Glebova and Ginty, 2004). Consistently, we recently estimated, in the mouse heart, one SN process every two CMs in the sub-epicardium (EPI), while the density appeared decreased in the sub-endocardial region (ENDO) (Pianca et al., 2019). Interestingly, such innervation pattern is different, in rat hearts, in which SN density is similar in the EPI and ENDO regions, while in rabbit, pig, and human, SNs are highly represented in the ENDO region (Pianca et al., 2019). Although the results presented above advanced the anatomical details in the study of heart innervation, they were not designed to define the three-dimensional (3-D) topology of the cardiac sympathetic network. To achieve this goal (and aligned with the broader definition of “optogenetics” i.e., the use of genetically-developed and cell-targeted optical sensors in biology), Freeman et al. used two-photon fluorescence microscopy, combined with computer-assisted image analyses, to generate, in small cardiac volumes from mice with SN-specific expression of Green Fluorescent Protein (GFP), the first 3-D reconstruction of the cardiac SN network (Freeman et al., 2014) (Figure 4C). While the inspection of thin heart sections suggested the coexistence of innervated and denervated CMs, the 3-D reconstruction of the neuronal tree revealed that SNs are represented in the heart with a density much higher than expected and suggested that each CM may be simultaneously contacted by several neuronal processes, all of which establish multiple neuro-muscular contacts at the regularly displaced neuronal varicosities. In keeping with the inclusive definition of opto-genetics, we took advantage from the co-expression of fluorescent *td-Tomato* in CMs of the α-MHC/ChR2-*td-Tomato* transgenic mice, to define in higher detail, in the intact 3-D tissue, the topology of interactions between SNs and CMs. To this aim, we applied a modified version of “CLARITY” tissue transformation (Chung and Deisseroth, 2013; Chung et al., 2013) and imaged with multiphoton microscopy both the red-native fluorescence of CM sarcolemma and SNs stained with anti-Tyrosine Hydroxylase (TH) antibody. This allowed demonstrate that, in the mouse heart, each CM is contacted by at least one SN and that, most frequently, a single CM receives multiple neuronal inputs from different processes (Figure 5). Notably, the 3-D reconstruction of the SN network in autoptic human heart blocks confirmed both such extent and the complexity of cardiac sympathetic innervation at microscopic level (Figure 6) (Pianca et al., 2019), although a fine characterization of the topology of autonomic innervation, throughout the whole myocardium, is still unavailable.

### A Lightly Inspection of Neurogenic Control of Heart Activity

Sympathetic neurons modulate the rhythm and force of heart contraction through the effects of NA activation of CM β-ARs (mainly β1-ARs), increasing intracellular [cAMP] which, in the SAN, directly enhances I_f_, increasing the rate of spontaneous automaticity, and, in ventricular CMs, enhances, mainly through PKA activity, the extent of Ca^2+^ turnover at each cardiac cycle (Zaccolo and Pozzan, 2002; Stieber et al., 2003; Rochais et al., 2004; Bers, 2008; Difrancesco, 2010). While these effects allow short-term adaptation of cardiac function to increased perfusional demand (e.g., exercise or emotional stress, intrinsic homeostatic mechanisms), we recently demonstrated that, in parallel, constitutive SN inputs are required to maintain physiologic size of the adult heart, acting on the equilibrium between protein synthesis and degradation, *via* the β2-AR/Akt/Foxo/ubiquitin ligase signaling pathway (Zaglia et al., 2012). That the SNS affects heart structure and signaling, in basal conditions, has been demonstrated by several studies (Ogawa et al., 1992; Kanevskij et al., 2002; O'Connell et al., 2003; Zaglia et al., 2012; Kreipke and Birren, 2015; Pianca et al., 2019). Taken altogether the results described above support the concept whereby the ANS controls cardiac function across a wide effect range covering both the subtle physiological variation in inter-beat interval (known as heart rate variability, HRV) and constitutive control of CM trophism, in basal conditions, to the urgent chronotropic and inotropic responses of the “fight-or-flight” reaction (Zaglia and Mongillo, 2017). In view of this evidence, one naturally wonders what mechanisms allow such flexible, yet accurate, control of heart homeostasis, and the unique potential of optogenetics was thus exploited to delve into neuroeffector mechanisms underlying cardiac SN function. With this scope, the Kay group generated in 2015, transgenic mice expressing ChR2 under control of the SN promoter, TH, which were used, in a proof-of-principle study, to optogenetically activate SNs and address the dynamics of the events linking β-AR activation to changes in cardiac function, in isolated Langendorff perfused hearts. In support of the method feasibility, photo-stimulation of cardiac neurons on the epicardial surface of isolated hearts caused positive chronotropic and inotropic responses, accompanied to reduced AP duration, and fast pacing elicited sustained arrhythmias, thus demonstrating the usefulness of the technique to address the effects of SNs in cardiac physiologic and pathologic mechanisms (Wengrowski et al., 2015). The neural influence on HR was also studied using optogenetics in *Drosophila melanogaster*, which, although phylogenetically far from mammals, represents an experimental model suited for genetic manipulation and allows easy molecular investigation of signaling mechanisms in both heart physiology and pathology (Malloy et al., 2017). Optogenetic actuation was, additionally, exploited in *in vitro* studies, both in primary and human Pluripotent Stem Cells (hPSC)-derived SNs, establishing “physical and functional connections and controlling the beating rate of cultured CMs” which, in turn, impacted on neuronal maturation, thus supporting that bi-directional crosstalk takes place between SNs and CMs (Oh et al., 2016). The retrograde transfer of molecules from heart to cardiac autonomic neurons was exploited for organ-targeted expression of opsins, by heart injection of adeno-associated virus, which allowed vagal neuromodulation of HR (Fontaine et al., 2021).

Altogether, these studies support the potential of optogenetics in the study of fundamental questions in neuro-cardiology, including the signaling dynamics underlying neuro-cardiac communication.

**Figure 7:**
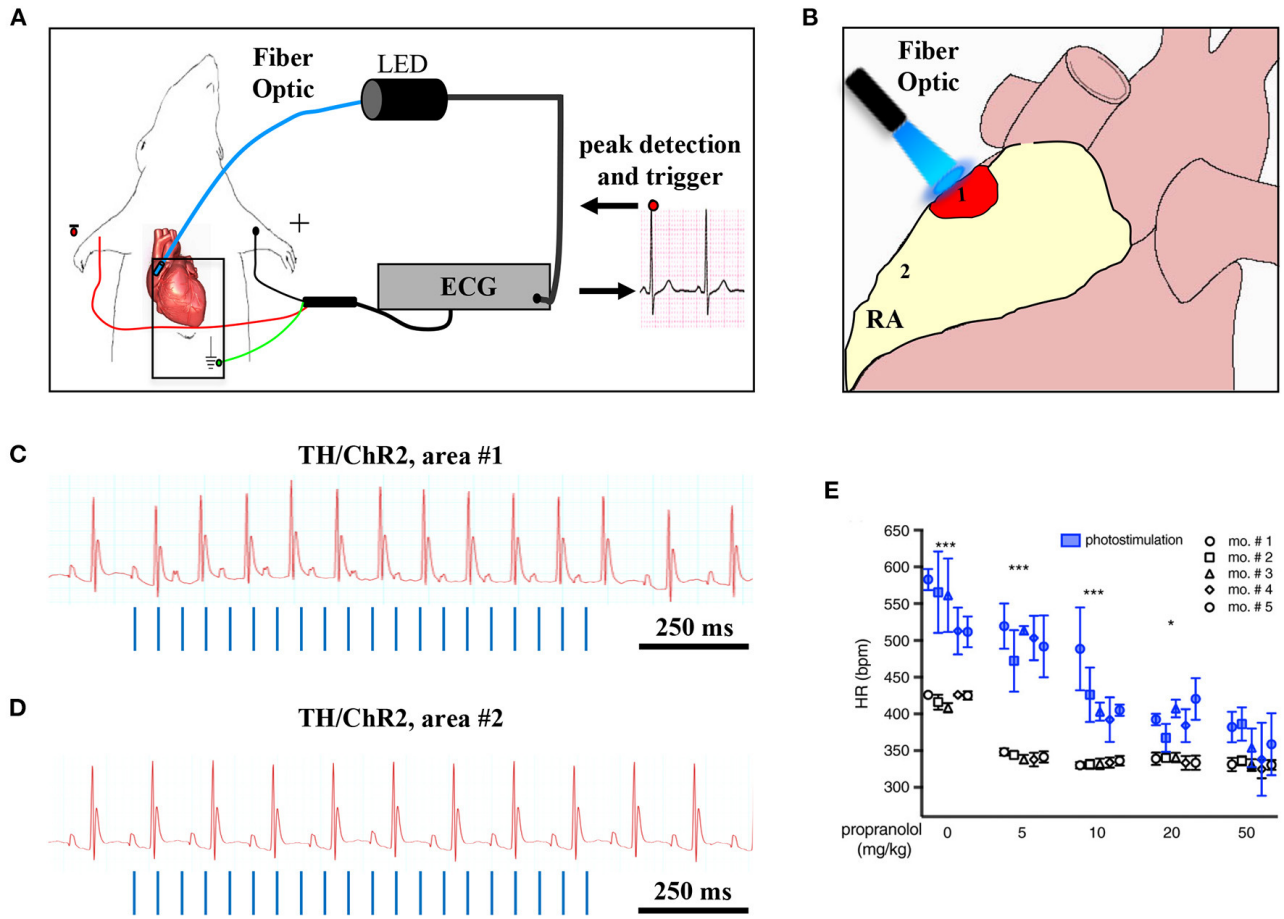
Optogenetic assessment of neuro-cardiac coupling *in vivo*. **(A)** Schematic illustration of the neuronal optogenetic set up used for right atrium (RA) illumination in open chest anesthetized mice. **(B)** Representation of the different photostimulated atrial regions (areas#1–2). **(C)** Representative ECG trace of the optogenetic experiment, showing positive chronotropic response upon photoactivation (blue lines) of the RA area#1 in TH/ChR2 mice. **(D)** Representative ECG trace showing unchanged HR upon illumination of RA area#2. **(E)** Dose-effect analysis of the treatment with the β-AR antagonist, propranolol, on the chronotropic response to neuronal photostimulation. Blue symbols identify responses to photostimulation, while the white ones show the effects of systemically delivered NA (***P < 0.001; *P < 0.05). Images modified with permission from Prando et al. (2018).

**Figure 8:**
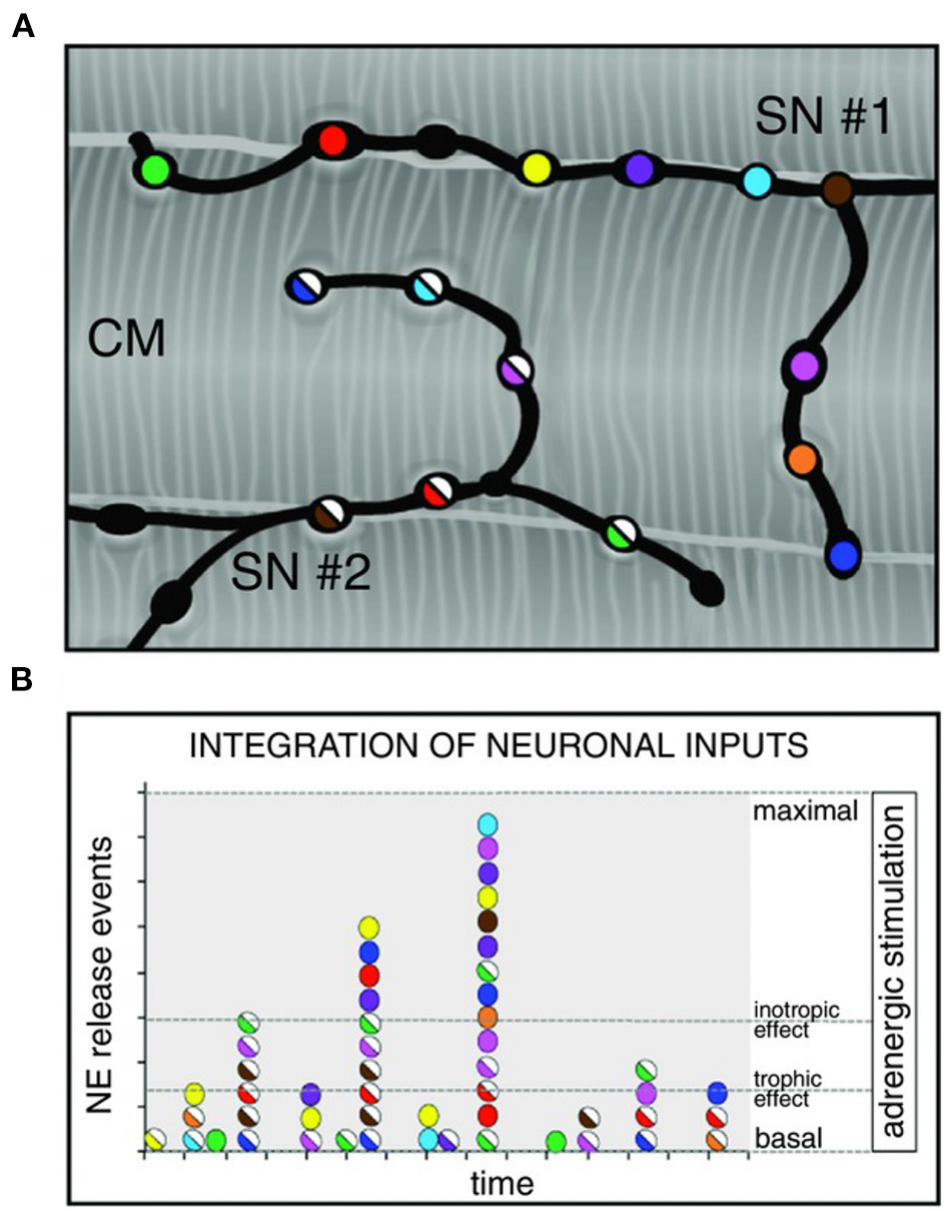
Integration of multiple neuronal inputs by the innervated cardiomyocyte. **(A)** Recent advancements in imaging cardiac sympathetic innervation demonstrate that each CM interacts with multiple contacts from the same neuronal process (each varicosity is highlighted by one color) and may simultaneously be innervated by different neurons (two neurons in the picture are represented by filled or half-filled circles, respectively). **(B)** We thus made the hypothesis that activation of increasing number of varicosities, and recruitment of more neuronal processes, may allow grading of the responses of target cells from basal to maximal activation, across a wide range of intermediate effects. Modified with permission from Zaglia and Mongillo (2017).

Although the rapid and efficient control of heart contraction, operated by SNs, exemplifies neurogenic regulation of organ function, the preferred view on sympatho-cardiac coupling in mainstream physiology aligns it more to a neuro-endocrine process than to a mechanism underlain by direct intercellular signaling. Activated SN processes, flanking CMs with no structured contacts, would simply discharge NA in the myocardial interstitium, at a concentration sufficient to activate β-ARs (Mann et al., 2014). However, such “reductionist” model does not explain convincingly the very fast (i.e., immediate) kinetics of heart response to SN activation, its efficiency and repeatability. With the aim to resolve these discrepancies, we used a combination of static (i.e., confocal immunofluorescence, electron microscopy) and dynamic (i.e., neuronal optogenetics combined to live imaging of cAMP or pharmacologic assays) optical methods to comprehensively address the neuro-cardiac interaction *in vitro* and *in vivo*. Co-cultures of SNs and CMs showed that neuronal varicosities establish structured intercellular contacts with CM membrane which define a diffusion-restricted, small volume intercellular signaling domain, allowing few molecules of NA to activate target cells at high concentration. These results indicated that direct synaptic communication occurred between SNs and CMs. To test whether this concept held true in the intact innervated heart, we reckoned optogenetics was the election method, and exploited the same set up, previously developed for cardiac optogenetics (Zaglia et al., 2015), to photo-stimulate the sympathetic efferents innervating the right atrium (RA), in living TH/ChR2-*td-Tomato* transgenic mice, while monitoring HR (Figures 7A,B). By combining photoactivation of sub-millimetric regions of the RA surface with pharmacologic inhibition assays using β-blockers, we demonstrated that SNs communicate with target CMs at directly interacting neurocardiac junctional sites, with fast kinetics and high [NA], favoring a “synaptic” rather than endocrine coupling model (Prando et al., 2018) (Figures 7C–E). Following a similar approach, Burton and colleagues applied optogenetics to interrogate the effects of SNs on “macroscopic” dynamics of a CM network *in vitro*, with the aim to identify arrhythmia mechanism in a cell-based system (Burton et al., 2020). In line with the results described in Prando et al., and those corroborated by the same group in 2019 (Pianca et al., 2019), this research promoted the concept that innervated CMs “can potentially receive NA simultaneously from multiple point sources, and integrate downstream the cumulative neuronal input at signaling level,” which support our speculation that direct neurocardiac coupling may explain how heart responses “can be graded across the wide physiological range of action of the cardiac SNS” (Zaglia and Mongillo, 2017) (Figure 8).

By taking advantage from the cell-type specificity of optogenetics, Kay's group progressed with the cognate of their previous work on SNs (Wengrowski et al., 2015), by targeting ChR2 to cholinergic neurons, and showed that PSN photoactivation, by inducing acetylcholine release, resulted in very rapid HR decrease (Moreno et al., 2019). In the same year, Rajendran et al. combined optogenetics to tissue clearing and physiological measurements, achieving higher anatomical details of vagal afferent and efferent fibers innervating the heart, thus further confirming the high complexity of heart neuronal circuitry, and advanced the understanding of the mechanisms involved in neurogenic control of HR (Rajendran et al., 2019).

Overall, these studies undoubtedly indicate that optogenetics is a tool suited “to provide new scientific insights into the structure and function of peripheral neural circuits” although “to disentangle neural control of autonomic physiology and enable a new era of targeted neuromodulation approaches” a combination of functional, imaging and physiologic approaches needs to be employed (Rajendran et al., 2019).

### Optogenetic Interruption of The Cardiac Arrhythmic Chaos

In normal hearts, the origin, sequence, and timing of cardiac activation repeat with identical characteristics at every heartbeat. The term arrhythmia, which means “out of rhythm,” indicates the alteration of any of the physiologic features of heart activation, including both faster and slower HRs, the abnormal origin of the cardiac activation wave, its faulty propagation throughout the different heart regions or, in its extreme manifestation, the erratic depolarization of heart cells. Due to the dramatic consequences which arrhythmias may have (i.e., SCD), the heart possesses a series of protective mechanisms to avoid uncontrolled cardiac activation, which include both cellular factors (i.e., refractoriness) and tissue properties, including the functional anatomy of the conduction system, and the electrotonic coupling of the CM network, which protects from initiation of cardiac contraction following stochastic activation of few cells (Hoyt et al., 1989; Rohr et al., 1997; Xie et al., 2010; Myles et al., 2012; Zaglia et al., 2015). As arrhythmias may develop when such protective properties are overcome, deeper understanding of these mechanisms is necessary to identify the cause and possible treatment of specific rhythm disturbances. In this context, the cell specificity, the elevated spatial and temporal precision of optogenetics have been exploited to determine the respective role of aberrant activation of either PFs or common CMs in the generation of ectopic cardiac activation foci and subsequent initiation of sustained arrhythmias (Zaglia et al., 2015). In addition, based on the advantages which optogenetics may offer, compared to conventional defibrillation (for a review see Entcheva and Kay, 2021), this technology allowed to successfully achieve cardioversion of atrial fibrillation in living mice (Bruegmann et al., 2018) and optogenetic cardioversion or defibrillation have been described, up to now, in a number of studies (Bruegmann et al., 2016; Scardigli et al., 2018; Uribe et al., 2018; Cheng et al., 2020; to name a few).

**Figure 9:**
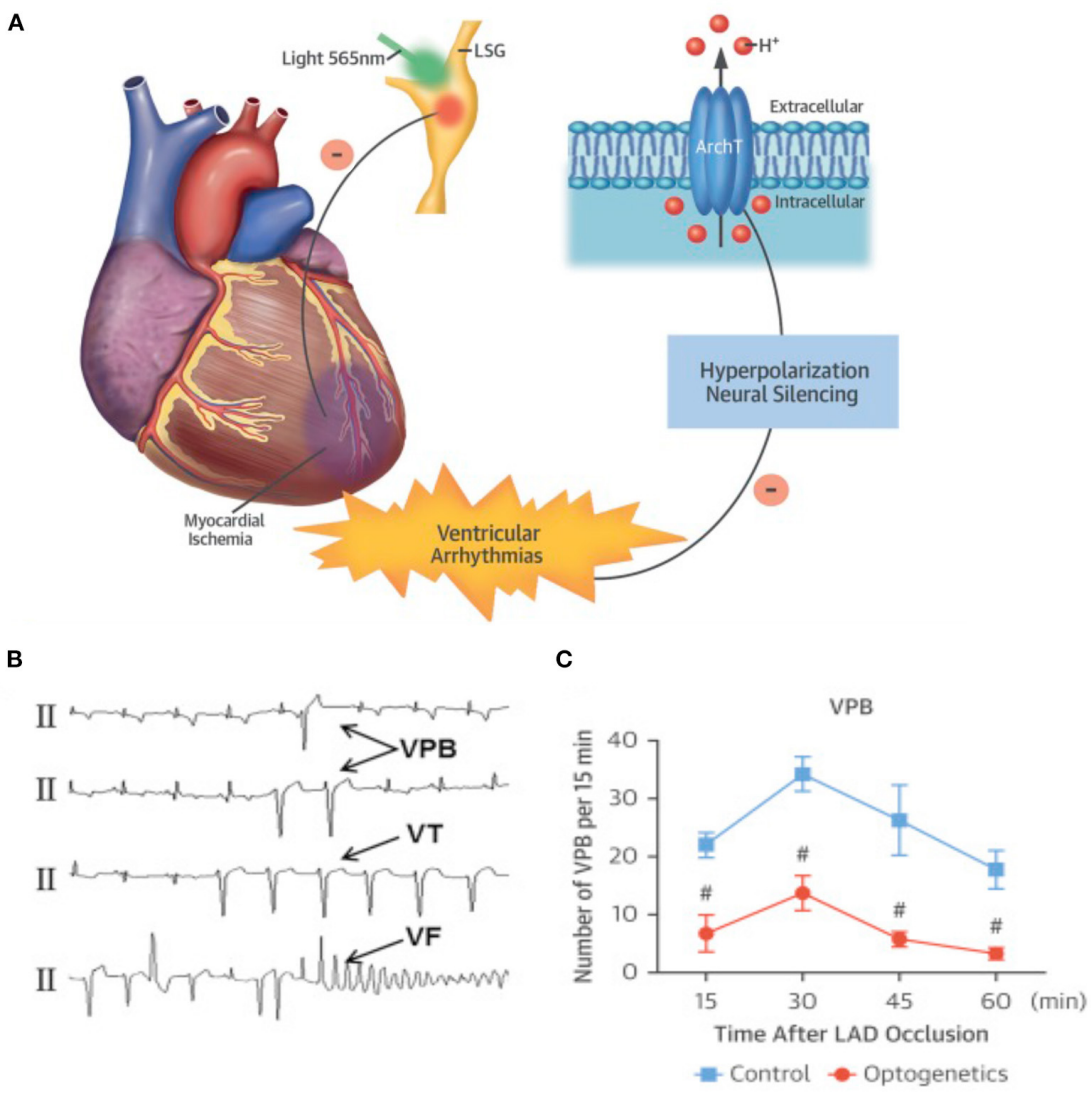
Optogenetic-based interruption of cardiac arrhythmias. **(A)** Schematic representation of the potential role of neuronal optogenetics in stopping ventricular arrhythmias. Photostimulation of ArchT with 565 nm light inhibits SNs, leading to significant decrease in arrhythmic events after myocardial ischemia. **(B)** Representative examples of ischemia-induced ventricular arrhythmias (Vas). **(C)** Quantitative analysis of the incidence of ischemia-induced VAs showed that optogenetic modulation significantly decreased the number of ventricular premature beats (VPBs). **(A–C)** Modified with permission from Yu et al. (2017) .

It is well-appreciated that the activity of SNs may increase arrhythmic vulnerability both through direct effects on single cell physiology and by reducing the “protective” myocardial properties at tissue level. In stress-dependent arrhythmogenic syndromes (e.g., CPVT, ACM), the link between SN activation and arrhythmia triggering has been shown both in single cells and experimental animals (Cerrone et al., 2005; Lehnart et al., 2008), and extensively supported by clinical evidence (Corrado et al., 1990, 2015; Amar et al., 2003; Collura et al., 2009; Shen and Zipes, 2014). In addition to the cellular effects of adrenergic stimulation, the concept that unbalanced NA discharge, by cardiac SNs, has arrhythmogenic potential has been suggested a few decades ago, and demonstrated since then in both structurally normal hearts of arrhythmic patients and ischemic hearts (Fukuda et al., 2015; Zipes, 2015; Gardner et al., 2016). The mechanism whereby regional heterogeneity of sympathetic inputs causes arrhythmia is indeed associated with AP dispersion, an electrophysiological state favoring ventricular arrhythmias. In support that regional differences in cardiac adrenergic stimulation promote arrhythmias, local injection of NA, in isolated perfused hearts, caused the onset of ectopic beats, which were not elicited by global perfusion with the same concentration of the catecholamine (Myles et al., 2012). It is foreseeable that optogenetics, allowing to deliver, with flashes of differently colored lights, the “go” (neuronal depolarization) or “stop” (neuronal hyperpolarization) signs to selected neuronal populations, appears ideally tailored to experimentally address the mechanisms correlating arrhythmias with SN function. The strong link between autonomic neuron input and cardiac arrhythmias, and the success of heart sympathetic denervation in reducing ventricular arrhythmias in sufferers from familial stress-dependent arrhythmogenic syndromes, have already fueled trust in the potential of autonomic neuromodulation of interrupting the electrical chaos of fibrillating arrhythmias, preserving the consonance of regular heart activation. This concept has been tested in the study by Yu and colleagues, showing that reversible optogenetic inhibition of the left stellate ganglion (LSG) activity increased electrophysiological stability and protected against post-ischemic ventricular arrhythmias. The authors expressed, by AAV9 infection, the photoactivated hyperpolarizing protein ArchT in the LSG of dogs and induced myocardial ischemia with left descending coronary artery ligation. Notably, photoinhibition of the transduced neurons significantly reduced the incidence of post-ischemic ventricular tachycardia (VT) and ventricular fibrillation (VF), providing “*proof-of-concept results of optogenetic arrhythmia therapy in a large-animal model*” (Yu et al., 2017; Entcheva and Kay, 2021) (Figure 9). The results of this study are in line with those obtained in 2012 by Mastitskaya et al. who demonstrated that photoactivation of vagal preganglionic neurons exerted protective anti-arrhythmic effect in a rat model of myocardial ischemia/reperfusion injury (Mastitskaya et al., 2012). Thus, the distinctive properties of optogenetics, and the flexibility of opsin expression in either selective intrinsic heart cells (i.e., CMs, PFs) or extrinsic modulators (i.e., SNs, PSNs) have opened the road to better understanding and novel treatment of arrhythmia mechanisms. Excitement and curiosity are in the air while expecting to see, as in the words of the poet Apollinaire, the *voice of light* tune cardiac rhythm.

## Can Optogenetics Uncoil the Intertwined Brain-to-Heart Connections?

The functional connection between the brain and the heart has been surmised several centuries ago, based on the appreciation that intense emotions and/or stresses reflected on the perceivable increase of HR and contraction force, frequently occurring in physiologic contexts, but could, at times, cause sudden heart arrest and death (Coote, 2007; Samuels, 2007). The neurogenic modulation of heart function, enacted by heart-innervating autonomic neurons, is in fact the integrated end-effect of numerous higher-order neuronal circuits which, from cortical, subcortical, and brainstem sites, converge on and regulate the activity of “motor” post-ganglionic autonomic neurons. Such hierarchical and multiplexed neuronal network allows the simultaneously active inputs of the somato-sensory system, sense organs, brain areas processing emotions, memory, and fear, to be marshaled into the system and reflect, with varied intensity and dynamics, on the degree of regulation of heart function.

**Figure 10:**
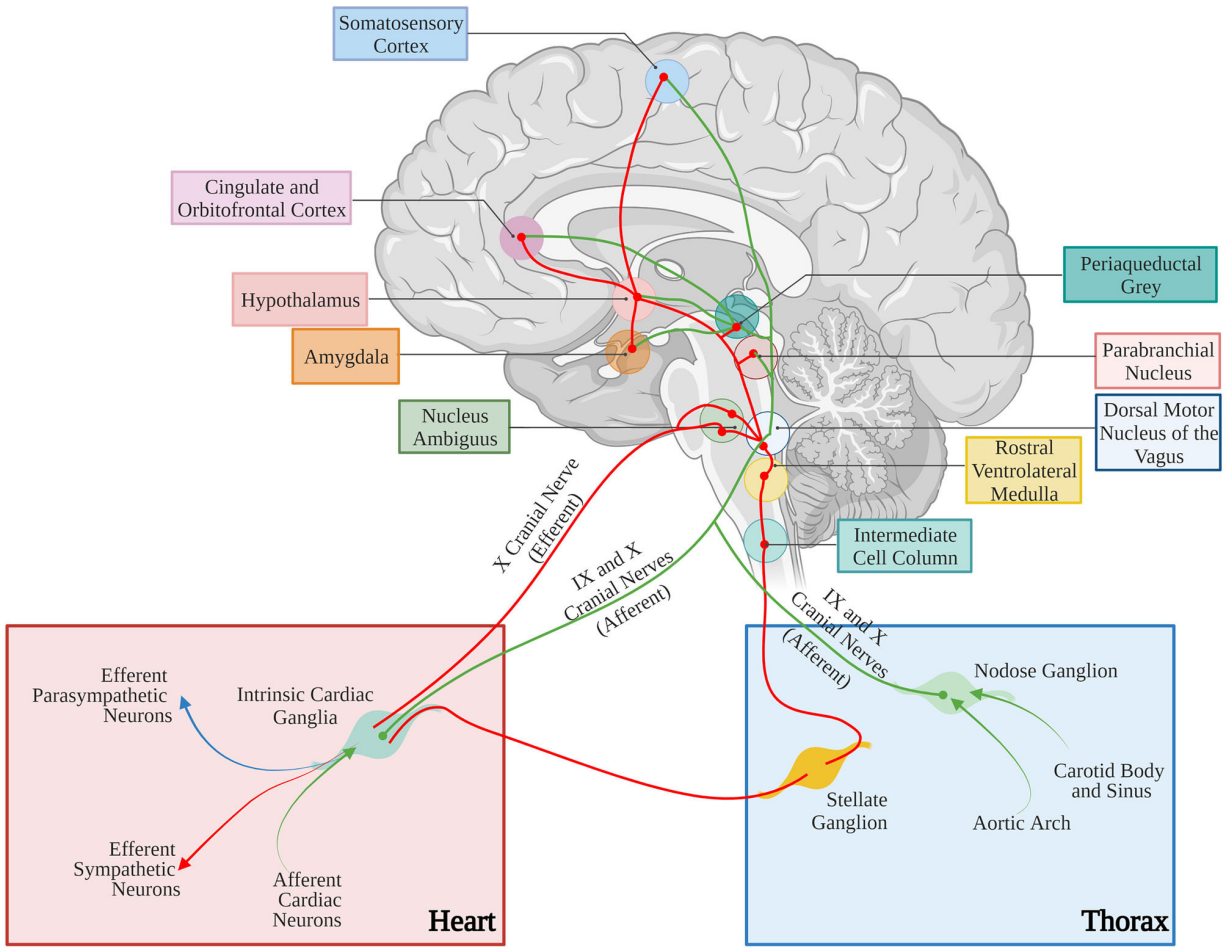
Neural control of the cardiovascular system. Afferent and efferent pathways are shown in green and red lines, respectively. The figure has been simplified to illustrate the major cortical, subcortical, and brain stem areas involved in control of the cardiovascular function. Most areas are interconnected. For anatomic details and physiological effects of the illustrated pathways, please refer to the text and the original article. Adapted with permission from Tahsili-Fahadan and Geocadin (2017). Adapted from “Anatomy of the Brain,” by BioRender.com (2021). Retrieved from (app.biorender.com/biorender-templates).

The anatomical basis of the “brain-to-heart” communication are, accordingly, grounded on complex neuronal circuitries, which lay the physical connection between the two organs (Figure 10). In its schematized representation, this neuronal network includes cortical and subcortical forebrain structures (i.e., cortex, insula, amygdala, and hippocampus, and their interconnecting systems), as well as the hypothalamus, the gray matter around the cerebral aqueduct (i.e., periaqueductal gray), and the nuclei of the parabrachial complex at the junction between the pons and brainstem (Augustine, 1996; Verberne and Owens, 1998). To further increase the complexity of this scenario, the central portion of the amygdala “receives inhibitory projections from the prefrontal and orbitofrontal area and is connected with the hypothalamus and the brain stem nuclei involved in control of the cardiac function and, thereby, seems to modulate the effects of emotional stimuli (especially negative emotions) on the heart” (see Tahsili-Fahadan and Geocadin, 2017 for a compendium). Furthermore, the hypothalamus is a transit point of information from the brain cortex and, in preclinical models, “the cardiac effects of stimulation of the lateral and anterior hypothalamus are preventable by sympathectomy and vagotomy, respectively” (see Tahsili-Fahadan and Geocadin, 2017 for a compendium). Although the detailed anatomy and function of each of these brain regions, and the modalities whereby they are connected to the gray matter of the spinal cord, where cell bodies of sympathetic ganglia neurons reside, is beyond the scopes of this review, this outlook serves to show the complexity of the extended brain–heart axis.

Understanding of the effects of brain areas described above on the heart, has mostly been obtained in animal models, and has conventionally used either electrophysiologic stimulation as trigger, and assessment of cardiac function as readout, or the destructive approach based on the analysis of functional effects of removal/lesion of specific brain regions. As an example, stimulation of the caudal and rostral posterior insular cortex in rats has been shown to decrease or increase HR, respectively, the latter effect elicited by activation of sympathetic outflow, as shown by its ablation with the β-blocker, atenolol (Oppenheimer and Cechetto, 1990). Combination of electrophysiological studies with surgical procedures (i.e., occlusion of cerebral arteries) has been employed to increase the depth of investigation. As example, monolateral occlusion of cerebral arteries supplying the insular cortex, in rats, prompted the “laterality hypothesis” whereby the right and left insula mediate increased sympathetic and parasympathetic tone, respectively, a phenomenon attributed to “the lateralized distribution of the baroreceptor units and processing of the emotions” (Oppenheimer et al., 1992; Zhang et al., 1998; Hilz et al., 2001) (see Tahsili-Fahadan and Geocadin, 2017 for a compendium).

Although these studies have undoubtedly advanced the understanding of neuro-cardiac anatomy and physiology, further progress requires higher precision and better definition of the function of cardiac wiring. Optogenetics is suited to be the “Rosetta Stone” enabling to decode the “brain–heart” talk, by systematically interrogating the effect of the different neuronal circuits impinging on heart function, in the living organism. Although such studies are still relatively scarce, we here present some relevant examples. Paul and colleagues, by combining multielectrode recording with optogenetic manipulations, showed that vasoactive intestinal polypeptide (VIP)-releasing neurons elicit diurnal waves of GABAergic input to cells of the paraventricular hypothalamus and ventral thalamus, while suppressing their activity during the mid to late hours of the day. Such circuit is involved in the circadian modulation of several physiologic functions, including HR (Paul et al., 2020). In addition, optogenetics elucidated how coordination between respiratory and cardiovascular functions is achieved. The combination of excitatory and inhibitory optogenetics with experiments of neuronal tracing, in rats, demonstrated that “preBötzinger Complex” (preBötC) neurons modulate cardiac PSN activity whilst excitatory preBötC neurons modulate sympathetic vasomotor neuron activity, resulting in HR and blood pressure oscillations in phase with respiration (Menuet et al., 2020). A similar approach, in rodents, was able to discern the mechanisms involved in arousal, wake-sleep cycle, and their impact on cardiovascular and respiratory control (Guyenet, 2006; Guyenet and Abbott, 2013; Smith et al., 2013; Dampney, 2016; Luppi et al., 2017; Saper and Fuller, 2017; Scammell et al., 2017; Del Negro et al., 2018; Benarroch, 2019).

In parallel to studies aimed to untangle central neuronal circuitries impacting on heart activity in physiology, optogenetics was also applied to complex neuro-cardiac pathologies, including e.g., the study of the bidirectional link between psychiatric disorders and heart diseases (Cheng et al., 2012).

Thus, the message emerging from this frugal overview of some examples on the theme, is that optogenetics may be a valuable tool to uncover the mysterious and multifaced “brain-to-heart” relationship. However, because of the incredible anatomical and functional complexity of the neuronal circuitries, which tie reason to feeling, optogenetics needs to be assisted in the task by other methodologies, including neuronal imaging, tissue clarification, behavioral, and functional studies.

## May Optogenetics Shed Light on the Unexpected “Heart-To-Brain” Correspondence?

The long consolidated understanding on the basic principles of heart physiology has somewhat concealed, or obscured, the increasing number of studies showing the extent of its nervous component.

The heart homes indeed a large series of different neuron types orderly networked in subsystems and circuits, receiving (as described above), integrating and sending neuronal impulses from and to the brain, justifying the proposed concept of “the little brain” of the heart (Armour, 2007, 2008). In addition to the efferent fibers of the two main branches of the ANS, the myocardium is densely innervated by sensory neurons, and the two systems are peripherally regulated by the interacting neurons residing in the INS. Cardiac sensory afferent neurons project to the CNS, transferring information of the chemo-, pain, and mechano-sensors which monitor the biochemical and mechanical state of the heart, mainly *via* release of substance P and calcitonin gene-related peptide (Hoover et al., 2008). The cell bodies of sensory neurons are found in spinal dorsal root ganglia, extracardiac intrathoracic, and intrinsic cardiac ganglia (Tahsili-Fahadan and Geocadin, 2017), and their ascending fibers take part to the IX and X cranial nerve and interact with the autonomic nuclei of brainstem, hypothalamus, amygdala, thalamus, and cerebral cortex (Tahsili-Fahadan and Geocadin, 2017) (Figure 10).

**Figure 11:**
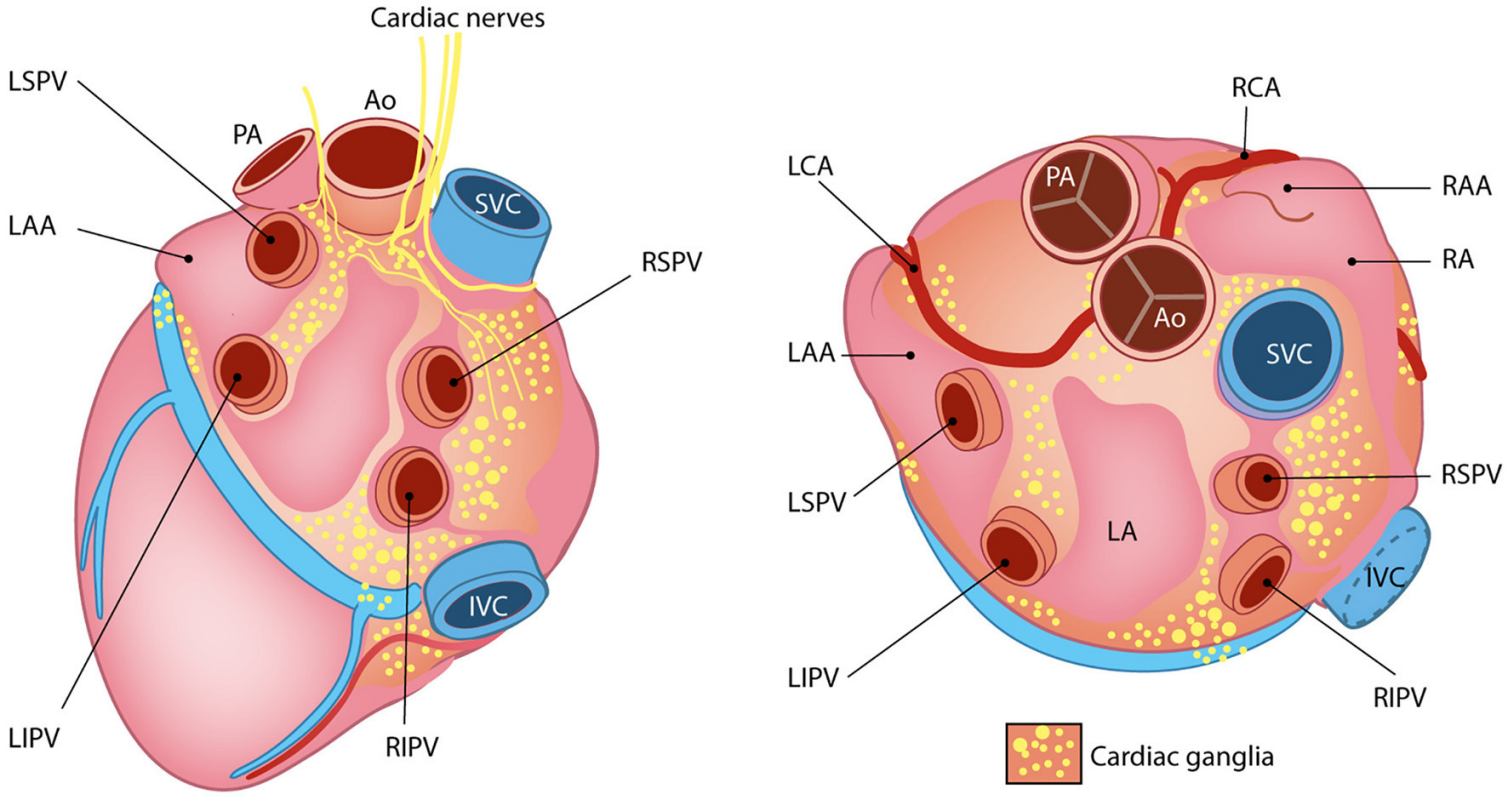
The little heart brain. Schematic representation of cardiac intrinsic ganglia. Ao, aorta; PA, pulmonary artery; LAA, left atrial appendage; LCA, left coronary artery; LIPV, left inferior pulmonary vein; LSPV, left superior pulmonary vein; RAA, right atrial appendage; RCA, right coronary artery; RIPV, right inferior pulmonary vein; RSPV, right superior pulmonary vein. Modified with permission from Wink et al. (2020).

The cardiac INS consists of a network of interconnected neuronal plexi which are located within specific heart regions, mainly the epicardial fat pads, and innervate the SAN (by the right atrial ganglionated plexi) and AVN (by the inferior vena cava–inferior atrial ganglionated plexi), as well as the pulmonary vein–left atrial junction (Armour et al., 1997; Tan et al., 2006; Tahsili-Fahadan and Geocadin, 2017) (Figure 11). Most cardiac intrinsic neurons are interneurons, mediating transmission of information within the resident ganglia. Such neuronal population is functionally connected to the efferent fibers of autonomic neurons in a two-way communication, which leads to a mutual influence on the nervous activity (Beaumont et al., 2013). Such complexity of cardiac neuronal circuitries and the notion that the heart sends collectively more signals to the brain than it receives, supports the recent findings attributing to the heart the capacity to perceive pain, independently from specialized brain centers (e.g., the thalamus), and may explain the involvement of the heart in regulation of central neurons of those regions (Alshami, 2019).

Interestingly, like the autonomic extrinsic efferents, remodeling of the cardiac afferents, and the INS may also occur in cardiac diseases, and have an impact on central neuronal circuitries (Armour, 2004). That a primary heart dysfunction negatively impacts on the correct function of higher neuronal centers is now supported by a large number of researches. As an example, it is a well-accepted notion that patients suffering from HF have increased incidence of stroke (mainly attributed to reduction in brain perfusion caused by cardiac contractile dysfunction), but also show cognitive decline, anxiety (Scherbakov and Doehner, 2018) and depression (Moradi et al., 2021). These may in turn, reflect on the activity of cardiac autonomic efferents, in a vicious cycle whereby cardiac pathology alters “heart-to-brain” communication, affecting “brain-to-heart” response, further compromising heart structure and function. In support of this concept, Koba and colleagues, have recently used optogenetics to show that sympatho-excitatory input from hypothalamic neurons, which project to the rostral ventrolateral medulla (RVLM), is enhanced after myocardial infarction (Koba et al., 2020). In addition, photostimulation of ChR2-expressing astrocytes in the RVLM of rats increased HR, through ATP-dependent enhancement of sympathetic activity. Notably, “facilitated breakdown of ATP in the RVLM attenuates the progression of LV remodeling and HF secondary to myocardial infarction” (Marina et al., 2013). The studies described above are based on the identification of central circuitries which, by receiving erroneous information from damaged myocardium, give wrong orders to the heart, thus further worsening its dysfunction. On the contrary, the use of neuronal optogenetics to decode the communication among the different cardiac neuronal circuitries and define their effects on heart function and homeostasis, although fascinating, has never been tested thus far, likely because of several technical limitations, discussed below in **Chapter 6**. Similarly, the use of cardiac optogenetics to dissect the mechanisms linking changes in myocardial contractile activity to alteration in CNS, which holds a high translational potential, has not been attempted. These represent rather unexplored territories to take a look at with neurocardiac optogenetics.

## Optogenetics in Neuro-Cardiology: Only a Delusion?

The studies described so far support that optogenetics is a very promising technique potentially very useful for decoding nerve circuits which bind, in a mutual dependence, the brain and the heart. However, peripheral neuron optogenetics (including that applied to neuro-cardiology) has a number of specific limitations with respect to its more common application in the field of neuroscience. Firstly, the popularity of the method in the study of central neuronal circuits has prompted the parallel development of technical means to deliver light (e.g., through lightweight fiber optics stably implanted in the skull), while similar devices usable for peripheral neuronal optogenetics are still in the early phase. This allowed to perform experiments in freely moving mice, often with immediate and easily quantifiable readouts (i.e., movement, behavior). On the contrary, interrogation of cardiac autonomic neurons has been performed, due to obvious anatomical constraints, in mice under anesthesia, which is well-known to interfere with neuronal activity (Yu et al., 2017; Prando et al., 2018; Moreno et al., 2019; Rajendran et al., 2019; to cite a few). In addition, when conscious animals are experimented, peripheral nerves in soft, moving tissues are easily injured during fiber-optic implantation, which may cause persistent irritation at the biotic/abiotic interface and constraint natural movements, thereby affecting or preventing free motions and increasing e.g., stress and anxiety (Zhang et al., 2019). This is particularly important when addressing the study of intra-organ (SN-CM) and inter-organ (brain–heart) neuronal communication. The scenario is further complicated by the technical difficulty of simultaneously operating optogenetic neuronal actuation, while monitoring cardiac readouts (e.g., ECG), in freely moving animals.

With these considerations in mind, understanding in full whether the potential of neuro-cardiac optogenetics is confined to science glamor, or if it is a guiding light in understanding neuro-cardiac pathophysiology, some additional steps should be overcome. Firstly, as the time of proofs-of-principle is now surpassed, optogenetics should be used when it represents the adequate solution to a scientific problem, and the research question is not based on the technique itself. Secondly, a broader consideration concerns how neurocardiac optogenetics can be translated to human research and therapy, which is inherently limited by the method specifics.

**Figure 12:**
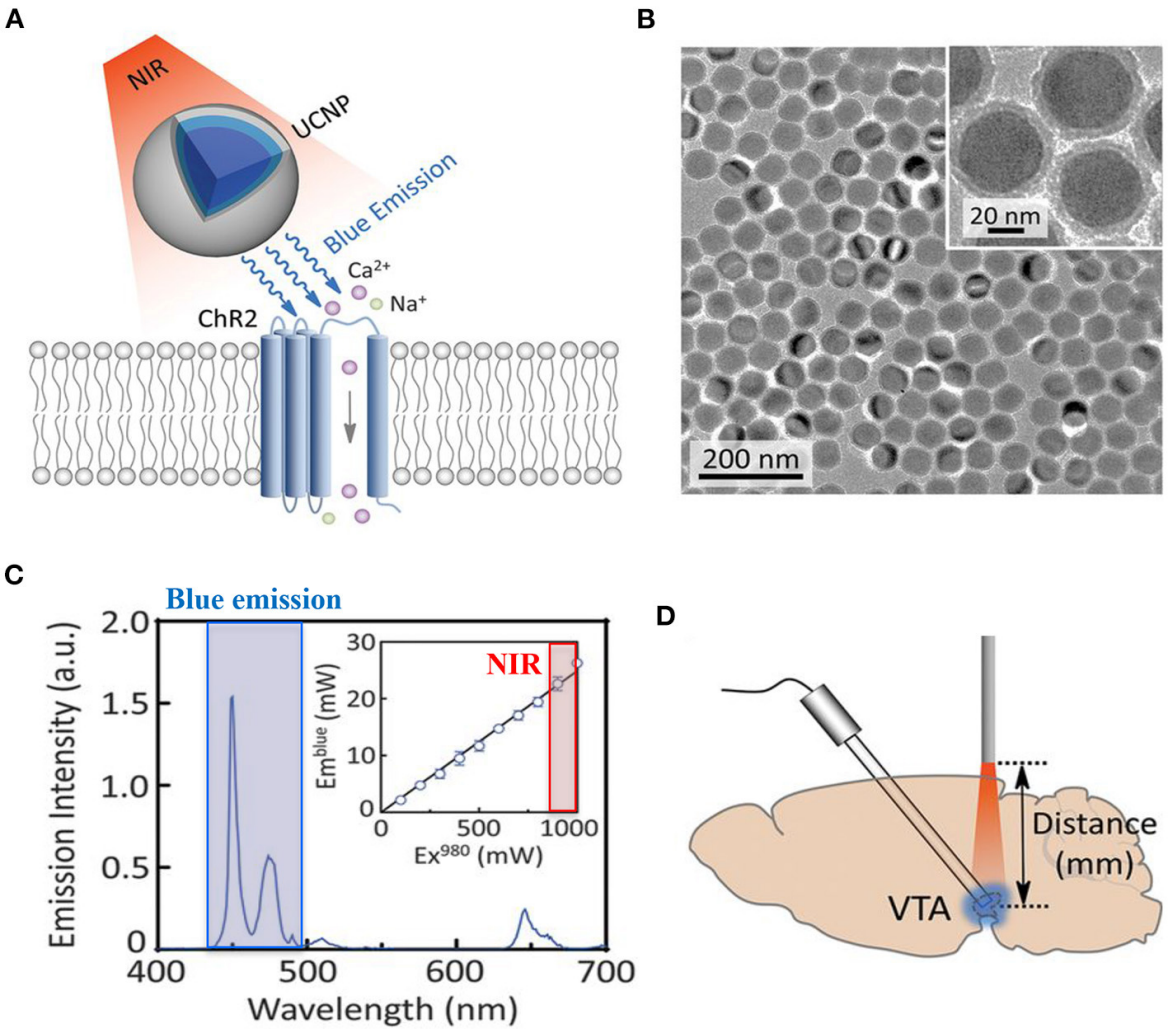
UCNP-mediated NIR upconversion optogenetics for deep brain stimulation. **(A)** Schematic principle of UCNP-mediated NIR upconversion optogenetics. **(B)** Transmission electron microscopy images of the silica-coated UCNPs. **(C)** Emission spectrum of the nanoparticles upon excitation at 980 nm. (Inset) Upconversion emission intensity of UCNPs as a function of excitation intensity at 980 nm. **(D)** Scheme of *in vivo* fiber photometry for measuring UCNP-mediated NIR upconversion in deep brain tissue. The tip of an optic fiber, transmitting NIR excitation light, was positioned at various distances from the ventral tegmental area (VTA) where UCNPs were injected. Modified with permission from Chen et al. (2018).

**Figure 13:**
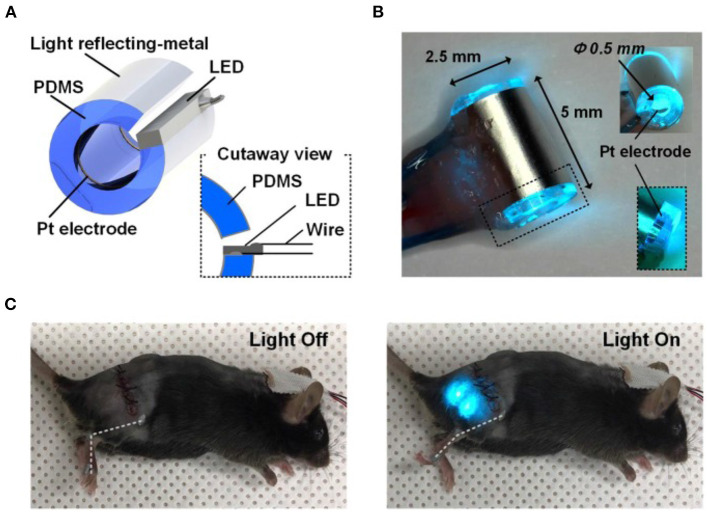
Optical nerve-cuff electrode for optogenetic stimulation of peripheral neurons in freely moving animals. **(A)** Overall schematic illustration of the optical nerve cuff electrode. **(B)** Picture of an active photo-stimulating device. **(C)** Pictures of a mouse implanted with the opto-cuff electrode in **(A,B)**. Examples of light off and light on states are shown. Modified with permission from Song et al. (2018).

When the road to optogenetics for a specific application has been undertaken, questions which guide the experimental design concern:

- **The choice of the most suited opsin:** from the first use of ChR2 in neuroscience (Boyden et al., 2005) to the time being, a large number of optogenetic actuators are now available, showing varied spectral and biophysical properties, which meet most of the experimental needs (www.optogenetics.org). The choice of the most appropriate opsin variant has to carefully be taken, and possibly confirmed in *in vitro* systems prior to investing in *in vivo* research.
- Due to its structure, **the myocardium is a dense highly diffractive tissue**, and as such, light in the visible spectrum has poor penetration capacity, thereby hindering minimally invasive translation of cardiac optogenetics to externally applied optical stimuli (Boyle et al., 2018). This has represented a crucial limitation for both cardiac and neurocardiac optogenetics, which has, so far, mostly been applied to surgically exposed organs (thus “canceling” the benefits of non-invasiveness). Solutions to overcome this hindrance are in the air, however. It is well-appreciated that near-infrared radiation (NIR) (780–1,100 nm) has deeper penetration capacity in tissues and minimal damaging effects, and as such, it is commonly used in deep tissue imaging (e.g., multiphoton microscopy) in both basic and clinical research, in different tissues (Gussakovsky and Kupriyanov, 2008; Nagarajan and Zhang, 2011; Wang et al., 2013). Although NIR is, at the time being, not suited for cardiac optogenetics, recent promising research has developed “upconversion nanoparticles (UCNPs),” absorbing tissue-penetrating NIR and emitting wavelength-specific visible light. Using this strategy, Chen et al. in 2018 demonstrated that UCNPs can serve as optogenetic actuators of transcranial NIR, and assist the photostimulation of deep brain neurons (Chen et al., 2018) (Figure 12). The combination of NIR laser with UCNPs methodology, after being successfully tested in neuroscience (Wang et al., 2017; Chen et al., 2018; Lin et al., 2018), has been applied to cardiac optogenetics, demonstrating the reliable and repeatable tissue-penetrating cardiac optical pacing *in vivo*, which demonstrated a convenient and less invasive way to use external radiation in optogenetic stimulation of cardiac tissue (Rao et al., 2020).
- In approaching a preclinical study focused on peripheral nerves, an inevitable task is **how to lightly control peripheral neurons within the tortuous nerve route**. Due to the infeasibility of implanting light delivery interface in peripheral nerves, in 2013 Towne et al. demonstrated the possibility to optically stimulate ChR2-expressing motor neurons, eliciting muscle contraction, in freely moving rats, thanks to the use of an implanted optical nerve-cuff around the sciatic nerve (Towne et al., 2013). From this first study, modern engineering has developed “optocuffs,” as optical peripheral nerve interfaces to achieve optogenetic control of peripheral nerves in freely moving mice (Michoud et al., 2018). Similarly, Song et al. invented a novel optical nerve cuff electrode allowing to simultaneously actuate and monitor neural signals, whose efficacy was tested in the sciatic nerve of Thy1:ChR2 mice (Song et al., 2018) (Figure 13). Since optogenetics study are often associated to pharmacological tests, recent interesting technological developments include the design of wireless, battery-free, fully implantable devices, capable of programmed delivery of localized optical, and/or pharmacological stimuli, using miniaturized electrochemical micropumps (Zhang et al., 2019). Organic light-emitting diode (OLED) able to adapt to curve surface and soft neuronal tissues have also been generated, which allow register the effect of optogenetics-based neuronal stimulation by magnetic resonance imaging (Kim et al., 2020). Here, we presented only some of the numerous examples of recently developed optical stimulation and recording devices (Samineni et al., 2017; Maimon et al., 2018) for a review see which, at the time being, have been successfully applied for optogenetic manipulation of motor and spinal nerves. Such devices are well-suited to instrument brain–heart connecting nerves and assess the effects of optically modulated neurons in freely moving animals, while recording heart rhythm or blood pressure changes (in telemetry implanted animals), or animal behavior. The development of similar tools apt to photoactivate heart-residing neurons in conscious animals, while monitoring central neuronal functions, would likely represent the tools of choice to investigate the reverse heart-to-brain axis.
- In preclinical studies, another fundamental aspect is **the choice of the most suited animal model**. At the time being the majority of optogenetics studies have been performed in mice, due to the availability of transgenic models. However, the use of larger animals (i.e., rats, minipigs, pigs, ovines), in which opsin expression may be achieved by viral infection (Yu et al., 2017; Booth et al., 2021), may increase the feasibility of *in vivo* analyses and the translability of the results to the human context.

Taken altogether, data described above indicates that all the ingredients are available to make of optogenetics a keystone to finally uncover, in depth and un-intrusively, the physiologic mechanisms underlying neurogenic control of heart function, as well as the brain–heart cross-regulation, thus bringing to the light novel targets for therapeutic intervention. Thus, optogenetics, although not directly used as a therapeutic strategy, may be potentiated to discover novel targetable players underlying neuro-cardiac disorders.

A different issue concerns the widely advertised therapeutic potential of optogenetics in neurocardiac diseases in humans. In general terms, the clinical applicability requires resolving two main obstacles: (**a**) optogenetics requires expression of an exogenous gene in neurons; (**b**) photo-actuation requires implantation of a light emitting source. For point (**a**), it has to be kept in mind that viral vectors suited to the use in humans, encoding ChR2, have been approved by FDA (NCT04278131; NCT03326336; NCT02556736; for details see www.clinicaltrials.gov), and their use for neuronal expression may thus be foreseen. As for (**b**), medical bioengineering may easily develop implantable and remotely powered devices similar to the ones already in use (e.g., pacemakers, ICD). For critical reading on the perspective applications of cardiac optogenetics in clinical settings (see Boyle et al., 2015; Entcheva and Kay, 2021).

## Conclusions

**Figure 14:**
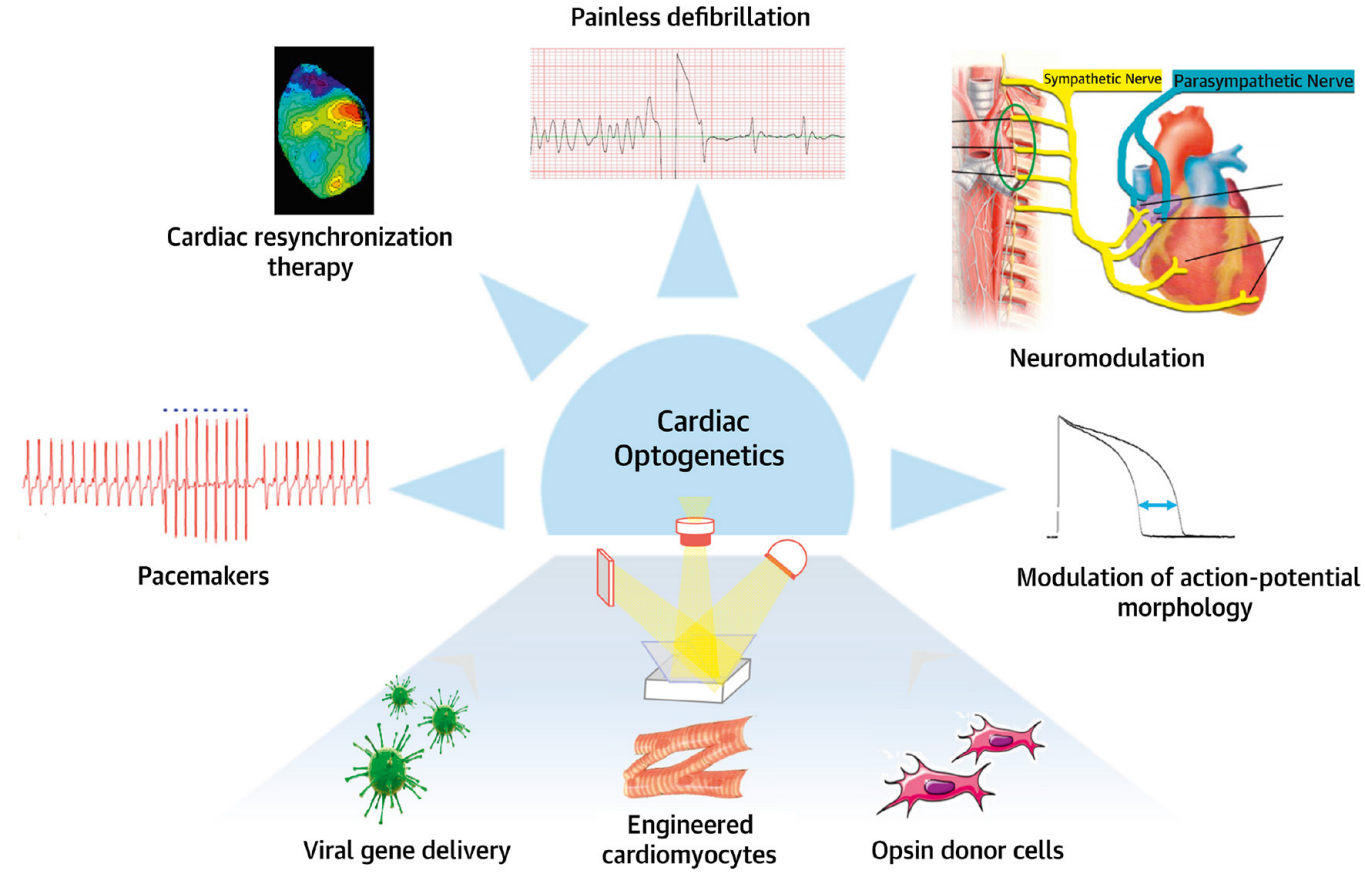
Potential applications of optogenetics. Potential clinical use of cardiac and neuronal optogenetics for heart rhythm control in neuromodulation, pacemaker, or/and anti-arrhythmic applications. Modified with permission from Gepstein and Gruber (2017).

This review was conceived and written with the brain and the heart to enhance the concept of brain–heart connection, which, even if supported by a gradually wider literature, is struggling to be an integral part of cardiovascular physiology and research. Here, we present an overview of current literature regarding the heart as a neuro-muscular organ, which not only is responding to hierarchically higher orders, but is capable of interpretation and decision-making, and reply back in the same language. Looking at the heart from this angle, we presented the contribution of optogenetics, treating the heart with light but not lightly, in untangling the “neuronal cardiac circuitries,” by unveiling the complexity of heart innervation, defining the mechanisms underlying neurogenic control of the heart in physiology and pathology, and perceiving the brain-to-heart communication. In assessing whether optogenetics is a truly revolutionary tool in cardiology, or only a mirage, we believe that, even if its applicability in the clinic may be limited to some pathologies, in basic research optogenetics can still give a lot, especially in a field as little explored, such as that of neuro-cardiology (Figure 14). However, as the constant synergy of the dialogue between the brain and the heart teaches us, to illuminate the dark sides of brain–heart connection, as well as of the intrinsic “cardiac little brain,” optogenetics must work synergistically with other methodologies, some traditional, others more innovative. Finally, we want to underly that this summary is by no means comprehensive, and we apologize to the many colleagues who contributed to the field, but have not been cited.

## Author Contributions

AS and NM contributed to manuscript and figure preparation. MM and TZ designed the review layout, drafted, and wrote the manuscript. All authors approved the final version of the manuscript and agree to be accountable for all aspects of the work, in ensuring that questions related to the accuracy or integrity of any part of the work are appropriately investigated and resolved, and that all persons designated as authors qualify for authorship and have been listed.

## Conflict of Interest

The authors declare that the research was conducted in the absence of any commercial or financial relationships that could be construed as a potential conflict of interest.

## Publisher's Note

All claims expressed in this article are solely those of the authors and do not necessarily represent those of their affiliated organizations, or those of the publisher, the editors and the reviewers. Any product that may be evaluated in this article, or claim that may be made by its manufacturer, is not guaranteed or endorsed by the publisher.

## Abbreviations

ACM: arrhythmogenic cardiomyopathy
ANS: autonomic nervous system
α-MHC: α-myosin heavy chain
AP: action potential
AVN: atrio-ventricular node
β-ARs: β-adrenoceptors
ChR: channelrhodopsin
CAN: central autonomic network
CNS: central nervous system
CPVT: catecholaminergic polymorphic ventricular tachycardia
CM: cardiomyocyte
DBH: dopamine-β-hydroxylase
DRG: Dorsal Root Ganglion
ECG: electrocardiography
ENDO: subendocardium
EPI: subepicardium
GFP: green fluorescent protein
HF: heart failure
HR: heart rate
HRV: heart rate variability
INS: intrinsic nervous system
LSG: left stellate ganglion
LV: left ventricle
ms: milliseconds
NA: noradrenaline
NIR: near-infrared radiation
NG: Nodose Ganglion
NPY: neuropeptide Y
OLED: organic light-emitting diode
PF: Purkinje fibers
PKA, PNS: parasympathetic nervous system; protein kinase A
preBötC: preBötzinger Complex
PSN: parasympathetic neuron
RA: right atrium
RV: right ventricle
SAN: sino-atrial node
SCD: sudden cardiac death
SCG: Sympathetic Cardiac Ganglia
SN: sympathetic neuron
SNS: sympathetic nervous system
TH: tyrosine hydroxylase
3-D: three-dimension
UCNP: upconversion nanoparticles
VIP: vasoactive intestinal polypeptide
VT: ventricular tachycardia
VF: ventricular fibrillation.
